# A zinc finger transcription factor enables social behaviors while controlling transposable elements and immune response in prefrontal cortex

**DOI:** 10.1038/s41398-024-02775-5

**Published:** 2024-01-25

**Authors:** Natalie L. Truby, R. Kijoon Kim, Gabriella M. Silva, Xufeng Qu, Joseph A. Picone, Rebecca Alemu, Claire N. Atiyeh, Rachael L. Neve, Jinze Liu, Xiaohong Cui, Peter J. Hamilton

**Affiliations:** 1 Department of Anatomy and Neurobiology, Virginia Commonwealth University School of Medicine, Richmond, VA USA; 2 Department of Biostatistics, Virginia Commonwealth University School of Medicine, Richmond, VA USA; 3 Gene Delivery Technology Core, Massachusetts General Hospital, Cambridge, MA USA

**Keywords:** Molecular neuroscience, Epigenetics and behaviour

## Abstract

The neurobiological origins of social behaviors are incompletely understood. Here we utilized synthetic biology approaches to reprogram the function of ZFP189, a transcription factor whose expression and function in rodent prefrontal cortex was previously demonstrated to be protective against stress-induced social deficits. We created novel synthetic ZFP189 transcription factors including ZFP189^VPR^, which activates the transcription of target genes and therefore exerts opposite functional control from the endogenous, transcriptionally repressive ZFP189^WT^. Following viral delivery of these synthetic ZFP189 transcription factors to mouse prefrontal cortex, we observe that ZFP189-mediated transcriptional control promotes mature dendritic spine morphology on transduced pyramidal neurons. Interestingly, inversion of ZFP189-mediated transcription in this brain area, achieved by viral delivery of synthetic ZFP189^VPR^, precipitates social behavioral deficits in terms of social interaction, motivation, and the cognition necessary for the maintenance of social hierarchy, without other observable behavioral deficits. RNA sequencing of virally manipulated prefrontal cortex tissues reveals that ZFP189 transcription factors of opposing regulatory function (ZFP189^WT^ versus ZFP189^VPR^) have opposite influence on the expression of genetic transposable elements as well as genes that participate in adaptive immune functions. Collectively, this work reveals that ZFP189 function in the prefrontal cortex coordinates structural and transcriptional neuroadaptations necessary for complex social behaviors while regulating transposable element-rich regions of DNA and the expression of immune-related genes. Given the evidence for a co-evolution of social behavior and the brain immune response, we posit that ZFP189 may have evolved to augment brain transposon-associated immune function as a way of enhancing an animal’s capacity for functioning in social groups.

## Introduction

Krüppel-associated box (KRAB) zinc finger proteins (KZFPs) represent an evolutionarily ancient class of protein [1, 2] and are the largest family of transcription factors (TFs) encoded in the mammalian genome [3]. KZFPs are characterized by a repressive N-terminal KRAB domain and a C-terminal poly-zinc finger array with sequence-specific DNA binding potential [4]. While growing evidence suggests that many KZFPs regulate genomic transposable elements (TEs) and control the expression of protein coding genes [5, 6], the complete gene-regulatory functions of individual KZFPs and how they contribute to organismal behavior remains poorly understood.

Earlier work employing weighted gene co-expression network analyses of RNA-sequencing (RNAseq) datasets of limbic brain areas from mice subjected to chronic social stress identified one member of the KZFP gene family, *Zfp189*, as the top regulatory transcript responsible for manifesting transcriptional networks in the prefrontal cortex (PFC) unique to stress ‘resilient’ rodents [7]. These resilient animals are able to endure chronic social stress without developing behavioral deficits in social interaction and other stress-related phenotypes. Both viral-mediated over-expression and CRISPR-mediated activation of *Zfp189* in PFC rescued social deficits in stress-susceptible mice [7]. Interestingly, in post-mortem tissue from human PFC (Brodmann area 25), individuals with major depression had lower expression of *ZNF189* (human ortholog) mRNA than matched controls [7].

Social dysfunction manifests in a multitude of neurological disorders including schizophrenia, autism spectrum disorder, and Williams syndrome [8] as well as in response to stressful life events which contribute risk for developing neuropsychiatric syndromes like major depressive disorder and post-traumatic stress disorder [9]. However, the brain molecular mechanisms that govern social behavior, and how these mechanisms are impacted in disease states, are not fully understood.

In order to interrogate the gene targets and specific social behaviors controlled by ZFP189, we developed novel synthetic ZFP189 TFs, each capable of exerting distinct forms of transcriptional control at in vivo ZFP189 target genes. We replaced the endogenous repressive KRAB moiety of wild-type ZFP189 (ZFP189^WT^) with the transcriptional activator VP64-p65-Rta (VPR) to create the novel synthetic TF ZFP189^VPR^. We reasoned that synthetically inverting the transcriptional control of ZFP189 would allow us to simultaneously disrupt and detect the in vivo transcriptional consequences of this poorly understood TF. We also removed the KRAB moiety entirely, generating a functionally inert control TF with no functional domain (NFD; ZFP189^NFD^). We combined intra-PFC viral delivery of these synthetic TFs with transcriptome profiling and behavioral assays to uncover the molecular mechanisms through which ZFP189 governs specific social behaviors. Viral delivery of both ZFP189^WT^ and ZFP189^VPR^ TFs to the mouse PFC resulted in maturation of dendritic spines on pyramidal neurons. ZFP189^VPR^-mediated inversion of natural ZFP189 transcriptional control within PFC precipitated pronounced social deficits, increased transcription of TEs and decreased expression of pathogen responsive immune genes. Among other social deficits, both socially dominant and subordinate ZFP189^VPR^-treated mice proved unable to maintain their established position in a social hierarchy, suggesting that ZFP189-regulated neurobiological mechanisms normally function to enable the social cognition necessary for participation in a social group. This body of work indicates that ZFP189 may tune an animal’s proclivity for social behavior by orchestrating a TE-regulated immune response in PFC cortical neurons to drive neuroplasticity and facilitate complex social behaviors.

## Results

### Synthetic ZFP189 transcription factors distinctly regulate gene expression and neuronal morphology

**Fig. 1:**
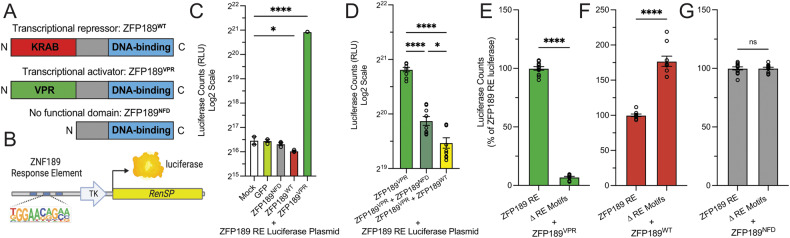
Synthetic ZFP189 transcription factors exert opposite transcriptional control at a luciferase target gene in vitro. **A** A cartoon representation of the three synthetic ZFP189 transcription factor (TF) proteins. **B** A cartoon of the ZFP189 DNA response element (RE) luciferase plasmid reporter target gene. TK is thymidine kinase gene promoter. *RenSP* is the LightSwitch™ luciferase gene. **C** Transient transfection of the ZFP189 RE luciferase plasmid and individual ZFP189 TFs in N2a cells reveals opposing transcriptional regulation of the luciferase target gene. Specifically, ZFP189^WT^ down-regulates and ZFP189^VPR^ up-regulates luciferase-driven relative light units (RLUs) relative to control conditions. One-way ANOVA followed by Bonferroni’s multiple comparisons test relative to mock transfection condition. *p-value < 0.05, ****p-value < 0.0001; *n* = 3 per condition. **D** Co-transfecting synthetic ZFP189 TFs of opposing function compete for regulation of the luciferase target gene. ZFP189^NFD^, and to a greater degree ZFP189^WT^, inhibit ZFP189^VPR^-mediated gene activation. One-way ANOVA followed by Bonferroni’s multiple comparisons test. *p-value < 0.05, ****p-value < 0.0001; *n* = 9 per condition. **E** Removing ZFP189 REs from the promoter of our luciferase target gene (∆ RE motifs) ameliorates the gene activation of ZFP189^VPR^. Data normalized to ZFP189 RE condition, by experiment. Two-tailed, unpaired Student’s *t*-test; ****p-value < 0.0001. *n* = 12 per condition. **F** Removing ZFP189 REs from the promoter of our luciferase target gene releases the gene repression of ZFP189^WT^. Data normalized to ZFP189 RE condition, by experiment. Two-tailed, unpaired Student’s *t*-test; ****p-value < 0.0001. *n* = 9 per condition. **G** Removing ZFP189 REs from the promoter of our luciferase target gene does not impact the functions of ZFP189^NFD^. Data normalized to ZFP189 RE condition, by experiment. Two-tailed, unpaired Student’s *t*-test; *p*-value > 0.05. *n* = 12 per condition.

To investigate the gene-regulatory functions of ZFP189, we employed a synthetic biology approach. We created three synthetic ZFP189 TFs: ZFP189^WT^ which is identical to the endogenous ZFP189 protein and contains the wild-type N-terminal KRAB domain and a C-terminal Cys_2_-His_2_ DNA-binding domain; ZFP189^VPR^ wherein the endogenous KRAB domain is replaced with the synthetic transcriptional activator VPR; and ZFP189^NFD^ in which the KRAB transcriptional regulatory domain is removed, which serves to control for any non-specific effects of the expression vector or the over-expressed ZFP189 DNA-binding domain (Fig. 1A). Since the DNA binding motifs of mouse ZFP189 have not been determined, we inserted the experimentally determined [10] DNA response element (RE) motifs of the human ortholog ZNF189, which is 92% identical to ZFP189 on the amino acid level, upstream of the thymidine kinase (TK) promoter to drive luciferase expression as a gene reporter for ZFP189 function (Fig. 1B). By co-transfecting mouse neuroblastoma Neuro-2a (N2a) cells with both the ZFP189 RE luciferase plasmid and our synthetic ZFP189 TFs, we observe that ZFP189^VPR^ induces robust luciferase activation, ZFP189^WT^ induces targeted gene repression, and ZFP189^NFD^ exerts no regulatory control (Fig. 1C). The null effect of ZFP189^NFD^ supports its use as the most appropriate control in future studies. By co-transfecting ZFP189^VPR^ alongside the other ZFP189 TF variants, we observe competition for regulation of luciferase expression. ZFP189^NFD^ impedes the gene activation of ZFP189^VPR^ likely via steric interference and competition for ZFP189 REs at the luciferase promoter, whereas ZFP189^WT^ further decreases ZFP189^VPR^ function via the additional action of the repressive KRAB domain (Fig. 1D). Lastly, to investigate the requirement of DNA-binding to putative ZFP189 REs in these TF functions, we excised the promoter ZFP189 RE motifs in our luciferase plasmid (Δ RE motifs) and compared the gene-regulatory functions of each ZFP189 TF. In the absence of ZFP189 DNA binding sites, we observe a 93% reduction in the gene-activating function of ZFP189^VPR^ (Fig. 1E). Conversely, we observe an increase in luciferase expression with ZFP189^WT^, indicating that the loss of ZFP189 binding disables repressive ZFP189^WT^ function (Fig. 1F). Interestingly, a similar effect is observed in N2a cells when no synthetic ZFP189 TF is co-transfected (Supp. Fig. 1) indicating the endogenous presence of ZFP189-like protein that functions similarly to our ZFP189^WT^. Lastly, the availability of ZFP189 DNA binding sites did not alter ZFP189^NFD^ function, further indicating its lack of transcriptional control (Fig. 1G). Collectively, these data illustrate that our synthetic ZFP189 TFs can specifically up- or down-regulate the expression of ZFP189 target genes via direct binding to genomic ZFP189 RE DNA motifs.

In the course of performing this research, it became apparent that N2a cells expressing synthetic ZFP189^VPR^ exhibited more complex cellular morphologies, including an increased number and length of cellular outgrowths (Supp. Fig. 2). This hints that the unique transcriptional control exerted by ZFP189^VPR^ regulates the cellular programs governing cell growth and differentiation in the mouse neuronal lineage N2a cells.

**Fig. 2:**
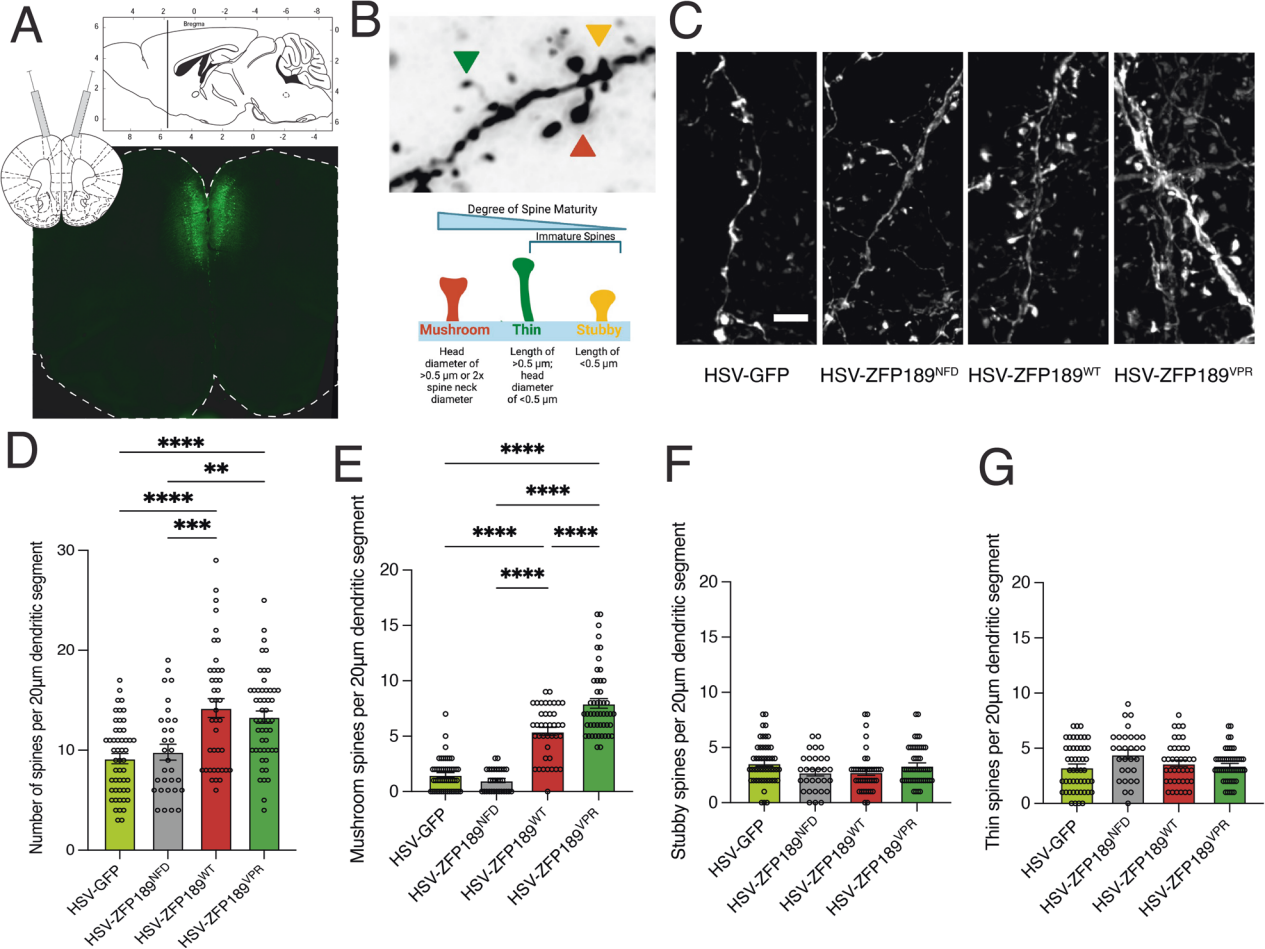
Viral delivery of synthetic ZFP189 transcription factors promote mature spine morphology in pyramidal neurons of the prefrontal cortex. **A** Representative image of viral targeting to the mouse prefrontal cortex (PFC). **B** Representative image of the three quantified spine morphologies (top) and the criteria for categorizing spines (bottom). **C** Representative pyramidal neuron dendritic segments from PFC tissues expressing GFP or one of the synthetic ZFP189 TFs. Scale bar is 2 μm. **D** Both HSV-ZFP189^WT^ and HSV-ZFP189^VPR^ (i.e., the transcriptionally functional ZFP189 TFs) similarly drive an increase in the number of spines per 20 μm dendritic segment. One-way ANOVA followed by Bonferroni’s multiple comparisons test. **p-value < 0.01, ***p-value < 0.001, ****p-value < 0.0001; *n* = 10 segments from 3-4 mice per condition. **E** HSV-ZFP189^WT^ and, to a greater degree, HSV-ZFP189^VPR^ increase the number of mature mushroom spines on transduced pyramidal neurons of the PFC. One-way ANOVA followed by Bonferroni’s multiple comparisons test. ****p-value < 0.0001; *n* = 10 segments from 3-4 mice per condition. **F** No viral treatment affects the number of immature stubby spines on transduced neurons. One-way ANOVA followed by Bonferroni’s multiple comparisons test. *p*-value > 0.05; *n* = 10 segments from 3-4 mice per condition. **G** No viral treatment affects the number of immature thin spines on transduced neurons. One-way ANOVA followed by Bonferroni’s multiple comparisons test. *p*-value > 0.05; *n* = 10 segments from 3–4 mice per condition.

To explore this possibility further in the PFC, the brain area in which we identified *Zfp189* [7], we packaged each of our synthetic ZFP189 TFs into herpes simplex virus (HSV) vectors for stereotaxic viral delivery to mice. Upon delivery to the PFC (Fig. 2A–C), we observed that both ZFP189^WT^ and ZFP189^VPR^ increase the density of dendritic spines on transduced pyramidal neurons. Specifically, ZFP189^WT^, and to a greater degree, ZFP189^VPR^ increase the density of mature mushroom spines relative to ZFP189^NFD^ and GFP controls, with no effect on the immature stubby or thin spines (Fig. 2E–G). These results indicate that both gene-activating and gene-repressive ZFP189 TFs affect neuroplasticity to drive dendritic spine maturity on PFC pyramidal neurons.

### Inverting ZFP189-mediated transcriptional control in prefrontal cortex induces social behavioral deficits

**Fig. 3:**
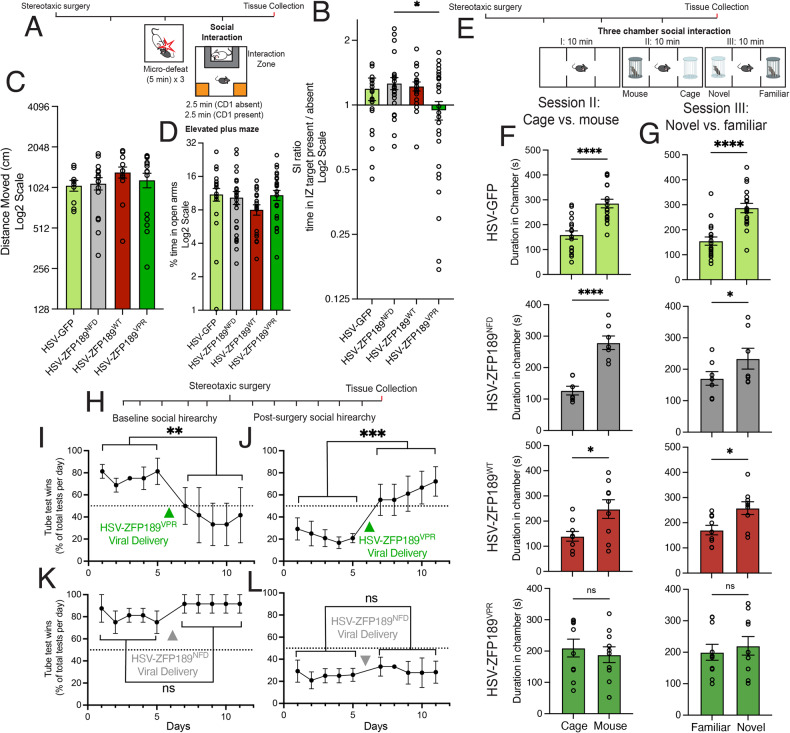
Inverting the natural transcriptional control of ZFP189 in the PFC with HSV-ZFP189^VPR^ impairs social function in mice. **A** Experimental timeline for viral delivery, micro-defeat, social interaction, and elevated plus maze testing. **B** Mice treated with HSV-ZFP189^VPR^ spend less time interacting with a CD1 social target mouse. Social interaction (SI) ratio quantifying the time test mice spent in interaction zone when social target mouse cage is empty relative to when the cage is occupied with novel CD1 mouse. Mice treated with HSV-GFP, HSV-ZFP189^NFD^, or HSV-ZFP189^WT^ prefer to socialize (SI ratio > 1), whereas HSV-ZFP189^VPR^ mice do not (SI ratio < 1). One-way ANOVA followed by Bonferroni’s multiple comparisons test. *p-value < 0.05; *n* = 21 mice (HSV-GFP) *n* = 18 mice (HSV-ZFP189^NFD^), *n* = 25 mice (HSV-ZFP189^WT^), *n* = 28 mice (HSV-ZFP189^VPR^). **C** No viral treatment condition affected locomotor behaviors in the SI test, measured in the first 2.5 min session when the target CD1 was absent. **D** No viral treatment condition affected time spent on the open arm of the elevated plus maze. One-way ANOVA followed by Bonferroni’s multiple comparisons test. *p*-value > 0.05. **E** Experimental timeline for three-chamber social interaction test. **F** In session II, when given the choice between interacting with an empty cage or a novel mouse, HSV-GFP, HSV-ZFP189^NFD^, and HSV-ZFP189^WT^ treated mice spend significantly more time interacting with the novel mouse, whereas HSV-ZFP189^VPR^ treated mice do not. Unpaired two-tailed Student’s *t*-test. ****p-value < 0.0001, *p-value < 0.05, ns *p*-value > 0.05. **G** Subjecting these same animals to session III, wherein test mice are given a choice between interacting with the caged mouse from the previous round (Familiar) or a novel mouse, HSV-GFP, HSV-ZFP189^NFD^, and HSV-ZFP189^WT^ treated mice again spend more time interacting with the novel mouse, whereas HSV-ZFP189^VPR^ treated mice do not. *p-value < 0.05, ns *p*-value > 0.05. *n* = 18 mice (HSV-GFP), *n* = 13 mice (HSV-ZFP189^NFD^), *n* = 10 mice (HSV-ZFP189^WT^), *n* = 11 mice (HSV-ZFP189^VPR^). **H** Experimental timeline for tube test. For five days, social hierarchy was determined by once daily head-to-head tube tests for all animals within a five-mouse cage. In each cage, either the two most socially dominant or the two most subordinate mice were delivered HSV-ZFP189 TFs intra-PFC whereas the remaining cage-mates were delivered HSV-GFP. Mice recovered for one day. For the following five days, the tube tests were repeated and wins for the HSV-ZFP189 TF treated mice versus all HSV-GFP treated cage-mates were recorded. **I** Delivering HSV-ZFP189^VPR^ to the PFC of previously socially dominant mice decreases the probability of winning a tube test to a rate of random chance (dotted line). Two-way repeated-measures ANOVA comparing main effect of wins pre- vs. post-viral intervention. ***p-value < 0.0001. *n* = 4 cages with two HSV-ZFP189^VPR^ mice per cage. **J** Delivering HSV-ZFP189^VPR^ to the PFC of previously socially subordinate mice increases the number of wins per day to a 50% chance of winning against an opponent. Two-way repeated-measures ANOVA comparing main effect of wins pre- vs. post-viral intervention. **p-value < 0.005. *n* = 6 cages with two HSV-ZFP189^VPR^ mice per cage. **K** Delivering HSV-ZFP189^NFD^ to the PFC of socially dominant mice does not change their tube test wins from pre-surgery performance. Two-way repeated-measures ANOVA comparing main effect of wins pre- vs. post-viral intervention. ns *p*-value > 0.05. *n* = 4 cages with two HSV-ZFP189^NFD^ mice per cage. **L** Delivering HSV-ZFP189^NFD^ to the PFC of socially subordinate mice does not change their tube test wins from pre-surgery performance. Two-way repeated-measures ANOVA comparing main effect of wins pre- vs. post-viral intervention. ns *p*-value > 0.05. *n* = 6 cages with two HSV-ZFP189^NFD^ mice per cage.

We next sought to uncover the behavioral consequences of dysregulating ZFP189-mediated transcriptional control in the PFC. As *Zfp189* expression in the PFC induces resilience to social stress-induced behavioral deficits [7], we first virally delivered GFP or the synthetic ZFP189 TFs to the PFC, subjected the animals to a sub-threshold social stress (micro-defeat), and performed social interaction and elevated plus maze behavioral testing (Fig. 3A) [7, 11, 12]. As expected, this mild stressor had no effect on the social behaviors of HSV-GFP, -ZFP189^NFD^ or -ZFP189^WT^ treated mice (Fig. 3B). Mice that received ZFP189^VPR^ in PFC showed social avoidance following the mild stressor (Fig. 3B). No viral treatment affected basal locomotion (Fig. 3C) or exploration of the open arm of an elevated plus maze (Fig. 3D). This indicates that inversion of the endogenous ZFP189 transcriptional control in PFC with ZFP189^VPR^ impairs social approach behaviors, but not basal or other anxiety-related behaviors.

To more completely interrogate the nature of ZFP189^VPR^-induced social deficits, we performed three chamber social interaction testing in test mice with no prior stress experience and treated intra-PFC with GFP or our synthetic TFs (Fig. 3E). When provided the opportunity to interact with either a novel mouse or empty cage, mice expressing PFC GFP, ZFP189^NFD^ or ZFP189^WT^ show a typical preference for social interaction (Fig. 3F). Mice expressing ZFP189^VPR^ show no preference for social interaction over investigating an empty cage (Fig. 3F). When this test is repeated with the choice between the mouse from the previous round (familiar mouse) or a novel mouse, ZFP189^VPR^-treated test mice again uniquely show no ability to discriminate, or preference for, social interaction with the novel mouse over the familiar mouse (Fig. 3G). In performing these same studies in female mice, ZFP189^VPR^ identically impairs social behaviors (Supp. Fig. 3), revealing that ZFP189 regulates these social behaviors in both sexes. When collectively analyzing these three chamber behavioral data by viral treatment, other treatment groups spend significantly more time with the mouse in Session II and the novel mouse in Session III than ZFP189^VPR^-treated mice (One-way ANOVA followed by Bonferroni’s multiple comparisons test; Session II: HSV-GFP vs HSV-ZFP189^VPR^ *p*-value < 0.0001, Session III: HSV-GFP vs HSV-ZFP189^VPR^ *p*-value < 0.0001). Collectively, these data further demonstrate that ZFP189^VPR^ behavioral control does not require prior stress experience, nor does it drive an active, anxiety-based social avoidance, as the ZFP189^VPR^-treated mice do not actively avoid the social chamber. Instead, ZFP189^VPR^ impairs an animal’s interest and/or awareness of social stimuli.

To understand the breadth of the detachment from social interaction induced by ZFP189^VPR^, we characterized the impact of ZFP189 dysregulation on an animal’s awareness and participation in prior established social hierarchy structures. We tested this by performing social dominance tube tests within each five-mouse cage before and after being manipulated with our synthetic ZFP189 TFs (Fig. 3H). Prior to viral-mediated gene transfer, we observe establishment and maintenance of social hierarchy amongst cage-mates. In socially dominant mice, characterized as consistently winning a majority of the tube-tests against familiar cage-mates, viral delivery of ZFP189^VPR^ to the PFC of socially dominant mice ablates consistent wins in the tube test versus cage-mates and the chance of winning regresses towards 50%, which could be explained by random chance in the binary win/loss outcome of a tube test (Fig. 3I). Conversely, in socially subordinate mice, characterized as losing the most tube tests against cage-mates, viral delivery of ZFP189^VPR^ to the PFC increases the consistent wins in the tube towards a random win/loss chance (Fig. 3J). Thus, both animals that were previously socially dominant or subordinate deviate from their cooperatively established position in a social structure and regress to a social performance that could be explained by random chance. Critically, identically performed experiments with dominant and subordinate mice treated with ZFP189^NFD^ do not show this deviation from their established social position (Fig. 3K-L), suggesting that this loss of hierarchical maintenance is specifically due to ZFP189^VPR^ PFC molecular function. This experiment indicates that ZFP189 dysfunction in the PFC removes the animal’s capacity to participate in cooperatively established social dominance hierarchies.

We next sought to investigate if these behavioral deficits could be explained by ZFP189^VPR^-driven deficits in social or object memory. We performed a five-trial social memory test wherein mice manipulated with our synthetic ZFP189 TFs were subjected to four sequential sessions of social interaction with the same social target mouse followed by a fifth session with a novel social target mouse. Mice in all treatment groups, including ZFP189^VPR^, significantly decreased time spent interacting with the increasingly familiar target mouse across sessions 1-4 and increased social interaction when the novel mouse was introduced in session 5 (Supp. Fig. 4). This indicates functioning social memory in all groups. As expected, animals manipulated with ZFP189^VPR^ interacted with the target mouse less overall (Supp. Fig. 4), reinforcing earlier observed deficits in social interactions. These data indicate that ZFP189^VPR^ does not ablate the entirety of an animal’s social function, as these animals are still able to discriminate and remember novel vs. familiar conspecifics. Lastly, we sought to ensure that ZFP189^VPR^ does not cause deficits in inanimate object recognition. We performed a novel object recognition test and discovered that mice in all treatment groups were able to recognize novel objects (Supp. Fig. 5). These data indicate the specificity of ZFP189^VPR^-induced behavioral deficits, in that it disrupts the higher-order social cognition, but not basic social and object memory.

### ZFP189 regulates transposable elements and immune genes in prefrontal cortex

**Fig. 4:**
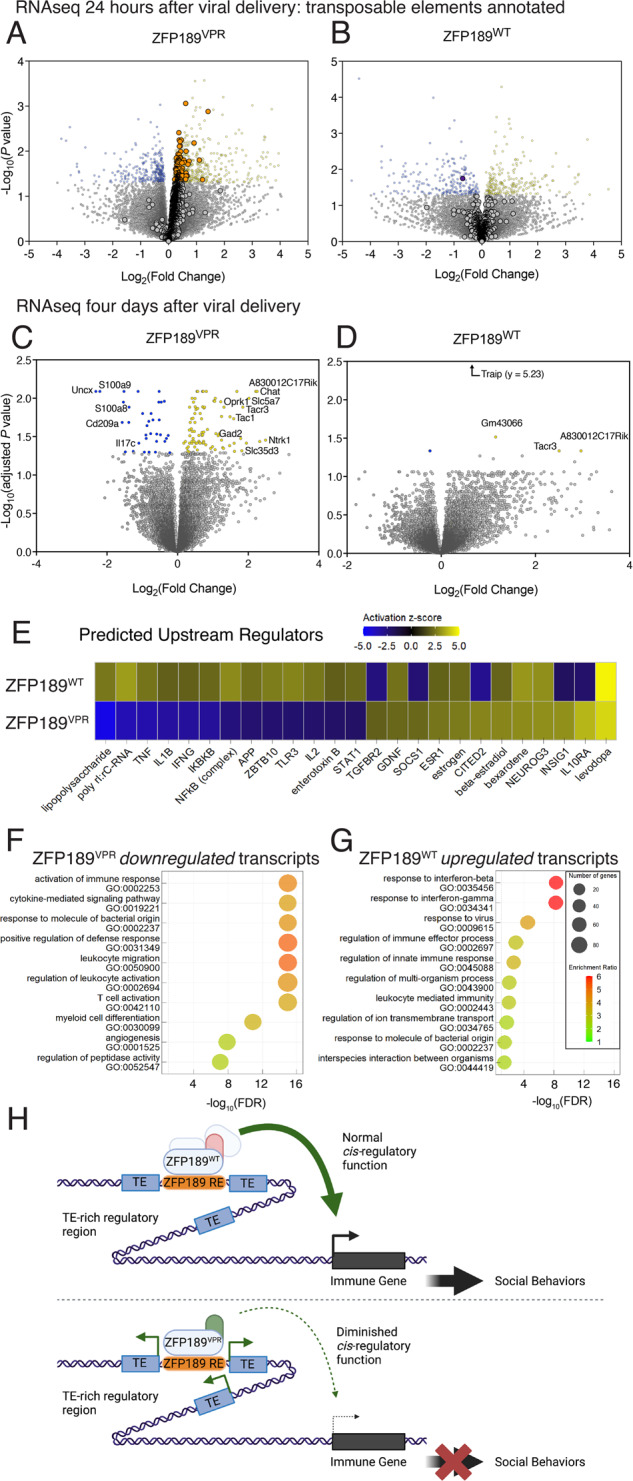
Synthetic ZFP189 TFs oppositely regulate transposable elements and genes related to immune function in prefrontal cortex. **A** Twenty-four hours after viral delivery to prefrontal cortex (PFC), RNAseq was performed and transposable elements (TEs) were annotated in the reference library alongside differentially expressed genes (DEGs). On volcano plots, TEs are represented as larger dots and DEGs as smaller dots. In HSV-ZFP189^VPR^ manipulated PFC, hundreds of distinct TE metagenes are up-regulated (orange dots), with no downregulated TEs. Differential TEs and DEGs generated relative to HSV-ZFP189^NFD^. Significance cutoff set to nominal *p*-value < .05. *n* = 5 microdissected PFC from individual mice (HSV-ZFP189^NFD^), *n* = 5 microdissected PFC from individual mice (HSV-ZFP189^VPR^). **B** In HSV**-**ZFP189^WT^ manipulated PFC, only one TE is detected as down-regulated relative to the control HSV-ZFP189^NFD^ (purple dot). Significance cutoff as in (**A**). *n* = 5 microdissected PFC from individual mice (HSV-ZFP189^NFD^), *n* = 5 microdissected PFC from individual mice (HSV-ZFP189^WT^). **C** Four days after viral delivery, another RNAseq was performed. Volcano plot depicting DEGs regulated by HSV-ZFP189^VPR^ PFC. Up-regulated DEGs are indicated by yellow dots, and down-regulated genes are indicated by blue dots. DEGs generated relative to our neutral HSV-ZFP189^NFD^ comparator. Significance cutoff set to 5% FDR corrected, Wald test adjusted *p*-value < 0.05. *n* = 7 microdissected PFC from individual mice (HSV-ZFP189^NFD^), *n* = 8 microdissected PFC from individual mice (HSV-ZFP189^VPR^). **D** Volcano plot depicting DEGs regulated by HSV-ZFP189^WT^ in PFC. DEGs generated relative to our neutral HSV-ZFP189^NFD^ comparator. Significance cutoff as in (**B**). *n* = 7 microdissected PFC from individual mice (HSV-ZFP189^NFD^), *n* = 5 microdissected PFC from individual mice (HSV-ZFP189^WT^). **E** Heatmap derived from Ingenuity Pathway Analysis (IPA) performed on complete DEGs lists from (**C**) and (**D**), weighted by fold change, reveals molecular factors whose function could explain the DEG expression profiles. HSV-ZFP189^WT^ vs. HSV-ZFP189^VPR^ regulated DEGs from (**C**) and (**D**) could largely be explained by increased (yellow) vs. decreased (blue) function of immune factors, respectively. **F**, **G** Gene ontology (GO) biological function terms enriched within the DEGs uniquely down-regulated by ZFP189^VPR^ (**F**) and from the DEGs uniquely up-regulated by ZFP189^WT^ (**G**) reveal that both DEGs lists are comprised of DEGs whose gene products participate in immune-related biological functions. **H** Cartoon representing how the normal function of ZFP189^WT^ can lead to the activation of immune-related transcripts and typical social behaviors. ZFP189^WT^ binds DNA motifs in TE-rich regions, represses the transcription of proximal TEs, recruits co-factors, and enables *cis*-regulatory action of this TE-rich region to enable the expression of immune genes in PFC, which, in turn, promotes social behaviors. Alternatively, ZFP189^VPR^ binds ZFP189 DNA motifs in TE-rich regions and activates the transcription of proximal TEs. This diminishes the *cis-*regulatory function of TE-rich regions and results in lowered expression of *cis-*regulated immune genes and impaired social behaviors. Cartoon made in Biorender.com.

The gene regulatory functions of the ZFP189 TF are poorly understood. To illuminate the potential molecular mechanisms by which ZFP189 controls social behavior, we performed RNAseq on micro-dissected PFC virally over-expressing synthetic ZFP189 TFs of opposing gene-regulatory function. To capture and identify direct ZFP189 gene targets, we performed RNAseq 24-h after delivering HSV-ZFP189 TFs to the PFC. HSVs deliver their *trans*-genes within hours [13], and we could both visualize HSV-delivered GFP within the PFC and could align sequencing reads to the HSV plasmid (not shown), indicating successful expression of our viral constructs at this early time-point. Given the major role that KZFPs like ZFP189 play in regulating the expression of TEs [5, 6], we hypothesized a potential role for TEs in this mechanism. We modified RNAseq reference libraries to annotate TEs and generated differentially expressed TEs and differentially expressed genes (DEGs) relative to the matched ZFP189^NFD^ condition. ZFP189^VPR^ activated the expression of hundreds of distinct TEs, with no TEs detected as repressed (Fig. 4A, differential lists in Supp. Table 1). ZFP189^WT^ had a more modest effect on the expression of TEs relative to control conditions, perhaps due to the endogenous expression of ZFP189 in the control tissue (Fig. 4B, differential lists in Supp. Table 2). This asymmetric up-regulation of TEs induced by ZFP189^VPR^ suggests that ZFP189^VPR^ binds immediately at TE-rich regions of DNA and activates the expression of proximal TEs.

We next sought to uncover the downstream consequence of this dysregulation of early ZFP189 TE gene targets. In a separate cohort, we generated DEGs four days post-viral surgery, the time-point at which we observed social deficits in Fig. 3. We observed no significant regulation of TEs at this later time point, therefore we only visualize DEGs on subsequent figure volcano plots. Further, in these datasets we were able to detect alternative splicing consistent with the inclusion/exclusion of the exon encoding the *Zfp189* KRAB moiety in ZFP189^NFD^ vs. ZFP189^WT^ comparisons (Supp. Fig. 6), which is a useful validation of the PFC expression and intended structural distinctions of our virally delivered TFs.

In mice treated with ZFP189^VPR^, we observe an up- and down-regulation of PFC DEGs (Fig. 4C, full DEG list in Supp. Table 3). Interestingly, ZFP189^WT^ produces fewer significant DEGs (Fig. 4D, full DEG list in Supp. Table 4). The lncRNA *A830012C17Rik* and the protein coding gene *Tacr3* were significantly up-regulated by both ZFP189 TFs, implying some converging function secondary to the demonstrated opposite direct gene regulation for these two ZFP189 TFs.

To more completely interrogate the PFC gene profiles regulated by our ZFP189 TFs, we employed Ingenuity Pathway Analysis (IPA) to identify and contextualize potential transcriptional regulators that would explain the expression patterns of our entire DEG lists from Fig. 4C-D. Strikingly, ZFP189^VPR^ - vs. ZFP189^WT^ -regulated DEGs could be attributed to decreased (ZFP189^VPR^) or increased (ZFP189^WT^) function of immune processes attributed to tumor necrosis factor (TNF), toll-like receptor 3 (TLR3), Interleukin 1β (IL1B), and Interferon γ (IFNG; IFN-ɣ), among other immune regulators (Fig. 4E, Supp. Fig. 7D-E). This result suggests a divergent impact on genes that participate in immune processes, with ZFP189^WT^ broadly activating, and ZFP189^VPR^ broadly inhibiting, the PFC expression of these immune-related genes.

To further explore the similarities and differences in function of our opposing ZFP189 TFs, we represented DEGs from the four-day timepoint in Venn diagrams (Supp. Fig. 7A-B) and employed gene ontology (GO) analyses. Transcripts uniquely down-regulated by ZFP189^VPR^ are related to activation of an immune response (Fig. 4F). Similarly, transcripts uniquely up-regulated by ZFP189^WT^ are also immune-related, with particular emphasis on the interferon adaptive immune system (Fig. 4G). In the commonly up-regulated transcripts, we observe that both ZFP189^WT^ and ZFP189^VPR^ converge in activating the processes related to synaptic function, including neuropeptide and neurotransmitter signaling (Supp. Fig. 7C). This complements the IPA analysis that identified L-DOPA as an active upstream regulator of both ZFP189 TF-regulated gene lists (Fig. 4E), as well as our earlier observation that any form of ZFP189-mediated transcription function in PFC potentiates synaptic maturity on pyramidal neurons (Fig. 2). These GO analyses also complement the IPA analyses to illustrate that the primary regulatory distinction between these ZFP189 TFs is to oppositely regulate the expression of transcripts involved in immune functions. Collectively, the use of synthetic ZFP189^VPR^ illuminates natural ZFP189 function. By dysregulating the natural function of ZFP189^WT^, we are able to ascertain the gene-regulatory functions of ZFP189, which is graphically represented in Fig. 4H and described in the Discussion.

## Discussion

By designing synthetic ZFP189 TFs capable of exerting opposing forms of transcriptional control in the brain, we are able to identify that ZFP189-mediated transcriptional regulation in PFC governs social behaviors in rodents. By inverting the natural, repressive gene-regulation of ZFP189^WT^ to gene-activation with ZFP189^VPR^, we observe that mice demonstrate pronounced deficits in social cognition with no other detectable behavioral abnormalities. In combining our transcriptional manipulations with RNAseq transcriptome profiling, we detect that ZFP189^VPR^ and ZFP189^WT^ oppositely regulate the expression of TEs and immune genes. By using the synthetic ZFP189^VPR^ in this research, we are able to dysregulate PFC TEs and implicate the importance of transcriptional control these PFC TEs in higher-order, group-based social behaviors. This work points to the possibility that the PFC function of ZFP189 has evolved to regulate these elements as a means of connecting brain immune response to social behaviors.

Our data are consistent with growing evidence to support the co-evolution of brain immune functions and the maintenance of proper social behavior. Since group-based social behaviors are critical to the survival of many species, and aggregation of organisms increases the likelihood of spreading pathogens, there may have been a co-evolutionary pressure for anti-pathogen immune signaling cascades to also regulate the neurobiological processes that drive an organism’s capacity for group-based social behaviors. Earlier reports have indicated that the function of adaptive immune signaling in PFC, specifically IFN-ɣ, drives prosocial behaviors [14]. It was demonstrated that IFN-ɣ deficient mice displayed social impairment without anxiety or motor deficits, which was reversible with administration of exogenous IFN-ɣ. Other research groups have uncovered that IL-17 signaling in neurons promotes social behaviors in mice [15, 16]. In our data, ZFP189^WT^ up- and ZFP189^VPR^ down-regulates the expression of genes whose products are involved in IFN-ɣ signaling cascades (Fig. 4E–G), and *Il17c* and IL-17 receptor genes are among the most down-regulated transcripts in the PFC of ZFP189^VPR^ treated mice (Fig. 4C).

It is appreciated that a major evolutionary driving force for the expansion of the KZFP TF family is to control the expression of TEs [5], including in the adult brain [3, 6], as a means of limiting their activity and potentially negative effects. In fact, it has been recently experimentally demonstrated that human KZFPs, including ZNF189, directly bind TEs [17]. We discover that ZFP189^VPR^ releases many TEs in the PFC (Fig. 4A), suggesting that ZFP189 normally binds and regulates TE-rich regions of the genome in brain, which is in agreement with this recent ChIP-seq dataset [17]. There is a robust and growing literature to indicate that TEs have evolved to act as *cis-*regulatory elements [18], particularly at enhancer regions for interferon and immune-related genes [19, 20]. We hypothesize that active transcription of these TE-rich gene loci, induced by our synthetic ZFP189^VPR^, incapacitates the *cis-*regulatory functions of these TE-rich DNA loci and inhibits the transcription of the predominantly immune-related *cis*-regulated genes. This potential mechanism would explain the gene-expression profiles we observe in our RNAseq studies and is represented as a graphic in Fig. 4H.

Since HSVs specifically infect neurons [13], our synthetic ZFP189 TF over-expression is restricted to PFC nerve cells. However, the PFC cell-types that harbor our observed DEGs are not possible to ascertain utilizing the bulk RNAseq approaches applied in this work. While IFN-ɣ is appreciated as originating from meningeal T cells, it is a soluble factor capable of acting upon IFN-ɣ receptors expressed on PFC microglia and neurons. Notably, IFN-ɣ signaling in cortical neurons is specifically implicated in regulating social behavior [14]. Further, IL-17 signaling through neuronally expressed IL-17 receptors is required for social behaviors [15]. Thus, we suspect that a majority of immune-related transcripts regulated by ZFP189 are localized within transduced PFC neurons, which would augment immune signaling and neuroplasticity within this cell type and consequently manifest the observed social deficits seen here. Indeed, we observe that both ZFP189^WT^ and ZFP189^VPR^ increase the density of dendritic spines and mature mushroom spines on transduced pyramidal neurons (Fig. 2E–G). This is in agreement with our RNAseq data, which shows that ZFP189^WT^ and ZFP189^VPR^ converge in activating gene expression related to synaptic function (Supp. Fig. 7C). Our previously published work has shown that *Zfp189* expression potentiates electrophysiological activity of nucleus accumbens neurons [21], which suggests that the spines we observe here may contribute to altered neuronal function in the PFC as well. Importantly, while ZFP189^WT^ and ZFP189^VPR^ exert opposing transcriptional regulatory control at direct ZFP189 gene targets (Fig. 1C), they are both transcriptionally active constructs and have broad transcriptional consequences downstream of their direct gene-regulation that diverge in their impact to immune genes (Fig. 4C–G) but similarly result in an increase in synaptic-related transcripts (Supp. Fig. 7C). We posit that either up- or down-regulation of ZFP189-regulated direct target genes results in broad transcriptional changes which acutely converge in an increase in spine growth. However, the functional consequences of these ZFP189-driven spines and the neural circuits in which they participate may be different across ZFP189 TFs. Further, here we present many lines of evidence that the net consequence of ZFP189^WT^ versus ZFP189^VPR^ function in the PFC results in largely opposing social behavioral outcomes (Fig. 3, Supp. Fig. 3). This implies in vivo neuronal morphology is sensitive to ZFP189-driven transcriptional control, yet the higher-order consequences of this neuroplasticity are dependent on the form of transcriptional control exerted by the ZFP189 TF.

We elected to employ our functionally inert ZFP189^NFD^ TF as the primary neutral comparator control group in these studies since we established that it exerts no regulatory control at a luciferase ZFP189 target gene (Fig. 1C) and therefore most appropriately controls for off-target protein interactions as a result of viral over-expression of our synthetic TFs. While we see a null effect for ZFP189^NFD^ which is comparable to GFP in these experiments, ZFP189^NFD^ likely has the capacity to function as a dominant negative by competing with any endogenously expressed PFC ZFP189. Importantly, this competition with endogenous ZFP189 would occur with any artificially over-expressed ZFP189 TF, including both ZFP189^WT^ and ZFP189^VPR^. We chose to control for these non-gene-regulatory consequences of viral delivery of a ZFP189 TF by using ZFP189^NFD^ as a control, in addition to our GFP control. Moreover, much of our prior research suggests that brain *Zfp189* gene expression is experience-dependent and is activated when the animal experiences chronic social stress or drug use [7, 21]. The animals in this study are naïve to these experiences, so the endogenous ZFP189 may not be highly expressed and subject to competition with the virally delivered ZFP189 TFs. Furthermore, it is unlikely that the observed effects in transcription of immune-related genes is a consequence of viral mediated gene transfer itself for a number of reasons. First, in all of our RNAseq analyses, we generated DEGs against the HSV-ZFP189^NFD^ condition, which is matched in terms of delivered viral particles and would therefore normalize out genes that respond to HSV infection per se. Second, many other studies have utilized identical viral-mediated gene transfer strategies, yet not observed an immune response in their transcriptomic analyses [22–24]. Most importantly, immune-related genes are bi-directionally regulated depending on the delivered ZFP189 TF, and are therefore unlikely to be a response to viral delivery itself. This supports the interpretation that our virally delivered synthetic ZFP189 TFs are themselves responsible for regulating our observed DEGs.

Our data also complement a growing body of research that has linked the brain expression and function of specific TFs, such as EGR1, to social behaviors in many organisms including songbirds [25, 26], fish [27], and rodents [28]. Importantly, EGR1 (sometimes called NGFI-A, KROX24, or ZIF268) is a zinc finger TF, whose brain expression has been noted to respond to social experiences and drive pro-social behaviors across phylogeny [25–28]. Given that ZFP189 is a member of a similar zinc finger TF gene family, it is possible that other structurally similar TFs, such as other KZFP TFs, regulate social behaviors via similar mechanisms of TE-regulated immune response. In support of this notion, the blind mole rat and the naked mole rat, two related organisms that differ widely in degree of TE regulation and immune responses [29–31] also represent extremes of social behaviors, ranging from highly solitary, in the case of the blind mole rat [32], to one of the few eusocial mammals with extremely complex and cooperative social structures, in the case of the naked mole rat [33]. Also, higher social status amongst macaques drives a proinflammatory response [34] via chromatin reorganization to expose TF DNA motifs [35]. Here, we observed that inverting ZFP189 gene-regulatory function in PFC removed the animal’s capacity to participate in cooperatively established group social structures, like social dominance hierarchies (Fig. 3H–L). This raises the intriguing possibility that eukaryotic brain TFs have co-opted the molecular mechanisms originally exploited by pathogens to influence social behaviors to facilitate pathogen transmission. It is possible that ZFP189 tunes the neurobiological mechanisms that facilitate social aggregation and group-based behaviors by regulating TE-rich regions of DNA, much of which is derived from ancient retroviruses [36], as a way of augmenting brain immune response and associated social behaviors, completely in the absence of external pathogens.

Lastly, ZFP189 was identified in an open-ended co-expression network analysis of RNAseq data across multiple limbic brain regions of mice that demonstrated phenotypic resilience to manifesting social deficits in response to chronic social stress [7]. *Zfp189* was the top key driver gene within the resilient-specific gene-network and *Zfp189* viral overexpression or CRISPR-targeted gene activation in PFC was sufficient to manifest the resilient-specific gene network and endow the animal with behavioral resilience to chronic stress, as measured in social interaction tests as well as other behavioral endpoints that do not involve social behaviors [7]. However, there was no indication that ZFP189 regulated the genes within the resilient-specific network via direct TF-gene interactions. Importantly, in post-mortem tissue from human PFC (Brodmann area 25), individuals with major depression had lower expression of *ZNF189* mRNA than matched controls. This raises the striking possibility that *Zfp189* expression in the PFC is biologically variable across individuals and sensitive to the experiences of the individual, such as the experience of chronic stress. This could mean that stress-induced social deficits observed in a number of neuropsychiatric syndromes are, in part, driven by PFC ZFP189 dysfunction and TE-associated dysregulation of immune functions. This research is a critical area for future investigation.

Here we present a novel approach in re-programming the functions of a poorly understood TF. By synthetically inverting the gene-regulatory function of ZFP189 in the brain, we discovered that ZFP189 tunes social function, regulates genomic TEs and immune response in PFC. This work uncovers novel connections between brain TFs, genetic transposons, immune response, and the social cognition necessary for group living.

## Methods

### Subjects

Male and female C57BL/6 J mice (8–10 weeks old) from Jackson Laboratories were used. Mice were group housed (5 mice/cage) on a 12 h light/dark cycle (lights on at 6am/off at 6 pm) with food and water freely available. All mice were used in accordance with protocols approved by the Institutional Care and Use Committees at Virginia Commonwealth University School of Medicine.

### Viral packaging

We de novo synthesized ZFP189^NFD^, ZFP189^WT^, and ZFP189^VPR^ and sub-cloned into HSV expression plasmids via ThermoFisher Scientific gateway LR Clonase II cloning reaction and Gateway LR Clonase II Enzyme mix kit (catalog number 11791-020 and 11971-100). Colonies were Maxiprepped (Qiagen Cat # 12163) and shipped to the Gene Delivery Technology Core at Massachusetts General Hospital for HSV packaging. Once packaged, aliquots were made and stored in -80 degrees C to be used in viral gene transfer through stereotaxic surgery.

### Viral gene transfer

Stereotaxic surgeries targeting the PFC were performed as previously described [7, 37]. Mice were anesthetized with I.P. injection of ketamine (100 mg/kg) and xylazine (10 mg/kg) dissolved in sterile saline solution. Mice were then placed in a small-animal stereotaxic device (Kopf Instruments) and the skull surface was exposed. 33-gauge needles (Hamilton) were utilized to infuse 1.0 μL of virus at a rate of 0.2 μL/min followed by a 5-min rest period to prevent backflow. The following coordinates were used to target the PFC: Bregma: anterior-posterior: +1.8 mm, medial-lateral +0.75 mm, dorsal-ventral −2.7 mm, 15 degree angle [7].

### Neuro-2a cell culture and transfection

*Mus musculus* Neuro-2a (N2a; ATCC^®^ CCL-131™) neuroblast cells were grown in adherent culture with 1:1 EMEM enriched / EMEM growth medium mixture (Quality Biological, #112-039-101; ATCC, #30 2003 or Corning, #10-009-CV) with 5% FBS (HyClone, #SH30071.03IH30-45) and 1.5% Penicillin Streptomycin (Gibco, #15140122) in a 37 °C and 5% CO_2_ Thermo Scientific HERAcell-150i CO_2_ incubator using aseptic techniques. N2a cells were maintained by passaging twice per week. One day before transfection, N2a cells of passage 60 or fewer were seeded in a 96-well plate at ~1.5 × 10^4^ cells/well. On the following day, the plasmid DNA of RE reporter (25 ng) was co-transfected together with ZFP189 TF expression vectors (100 ng or equal molar weight) on 80% confluent wells in triplicate for all treatment conditions with QIAGEN Effectene Transfection Kit (#301427) according to manufacturer instructions. Each reaction was carried out in 10 µl of buffer EC, 0.3 µl of Enhancer and 1 µl of Effectene, that was diluted to 100 µl in the medium before applying to the cells. The corresponding empty reporter vector RL (LightSwitch, #S990005) and empty expression vector GFP (p1005gw ∆*CCDB*) were used as background controls. The plates were centrifuged for 7 min at 2000rpm for higher transfection efficiency and were incubated for 1–3 days by covering with a Breathe-Easy sealing membrane only (Sigma-Aldrich, # Z380059-1PAK). All assays were performed on Day 1-3 following transfection. In experiments, each n corresponds to a distinct well, and experiments were replicated across 2-3 transfection reactions performed on different days.

### Luciferase assay

To assess the cell viability and toxicity, on day 2 following GFP reading, we conducted a Luciferase Assay Report using the CellTiter-Glo 2.0 Assay (Promega #G9242) and Renilla luciferase assay system (Promega #E2820). Relative Luminesce Unit (RLU) was measured by the BMG-Omega plate reader using the Luminescence endpoint program with gain 3432.

### N2a morphology assessment

The plasmid DNA of viral expression vectors (0.1-0.3ug) were transfected into N2a cells as described above in triplicate. The cell images of 48 h were captured in 0.1 µg transfection by a Bio-Rad ZOE_TM_. Fluorescent Cell Imager, followed by the GFP FI readout. For quantitative analysis of N2a morphology, we used Image J (FIJI) software to quantitate the number and length of each protrusion. We used the free-hand line tool to trace each protrusion with the set scale of 0.71pixel/µm corresponding to the 100 µm scale bar on each Fluorescent Microscopy photo. We then used the ROI manager measure of lengths to calculate averages. To further analyze our protrusion count and average protrusion length data, data from triplicate transfection reactions were averaged and normalized with the GFP readout. Standard deviation calculations of GFP readout and RLU measurements were used for error bars, respectively.

### Microdefeat stress

CD1 retired breeder mice were screened for aggressive behavior as previously described [38] A C57BL/6 J mouse is subjected to subthreshold levels of social defeat that consist of three 5-min defeat sessions given consecutively on a single day with 15 min of rest between each session. In each defeat session, a C57BL/6 J mouse is placed in the home cage of a single-housed CD1 mouse, where the CD1 mouse attacks the C57BL/6 J mouse [38].

### Social interaction (SI)

Behavioral testing was performed 24 h after exposure to sub-threshold microdefeat stress. Social interaction was performed as previously described [38] C57BL/6 J mice were placed into the open arena (43 × 43 × 43 cm) with an empty wire cage (10 × 5 × 30 cm) at one side (interaction zone). Mice were given 2.5 min of habituation to explore the arena and then removed from the open arena. A novel CD1 aggressor was then placed within the wire cage (interaction zone) and the C57BL/6 J was placed back into the open arena and another 2.5 min were recorded. The arena was cleaned with 70% EtOH before a new C57BL/6 J mouse was placed in the arena and before the first trial. Data were analyzed as time spent in the interaction zone without the aggressor compared to time spent within the interaction zone with the aggressor present. The data was then calculated into SI ratios by dividing the time spent in the interaction zone with the target mouse present by the time spent in the interaction zone (IZ) with the target mouse absent (SI ratio = time in IZ target present / time in IZ target absent.) Total distance moved (locomotion) was recorded and evaluated when the target was absent. Behavioral analyses were performed automatically by video tracking software (Ethovision Noldus) [39]. All behavioral tests were performed in a specified behavioral suite under red light.

### Elevated plus maze (EPM)

The EPM apparatus is constructed of black Plexiglas and consists of two open arms (33 × 6 cm) and two closed arms (33 × 9.5 × 20 cm) facing connected by a central platform (5 × 7 cm) [40]. The maze was kept elevated 63 cm above the floor. A C57/BL6J mouse was placed individually in the right-side closed arm facing the center of the plus-maze. Placement of all four paws into an arm was registered as an entry in the respective arm. The time spent in each arm was recorded during the 5 min EPM test. The platform of the maze was cleaned with 70% EtOH following each trial and before the first trial. The percentage of time spent in the open arms was calculated (time spent in open arms/300 s * 100 = % time spent in open arms.) Behavioral analyses were performed automatically by video tracking software (Ethovision Noldus) [39]. All behavioral tests were performed in a specified behavioral suite under red light.

### Three chamber sociability and social novelty test

The three-chamber test was used to assess sociability and interest in social novelty or social discrimination. The testing arena consisted of three adjacent chambers (each 41 × 21 × 41 cm) separated by two clear plastic dividers and connected by open doorways (5 × 9 cm). The test consisted of three 10-min sessions. The subject mouse begins the session in the middle chamber. In the first session, subject mice were allowed to habituate to the arena and freely investigate the three chambers. In the subsequent sociability session, a novel C57BL/6 J same-sex mouse (target mouse 1) was placed in a cylindrical cage (20 cm height × 10 cm diameter solid bottom; with clear bars spaced 2 cm apart) in one of the side chambers and another identical inverted empty cup was placed in the other side chamber. In the social novelty session, the empty pencil cup was removed and replaced by target mouse 1 in a new pencil cup. A second novel C57BL/6 J same-sex mouse (target mouse 2) was placed at the previous position of target mouse 1 under a new pencil cup. Target mice were the same sex and age as the subject mice. The chamber and cages cups were cleaned with 70% EtOH between animals and before the first animal. Time spent in each chamber was recorded. Sociability was measured by comparing the time spent in the chamber with a novel mouse vs an empty cup. Social novelty was measured by comparing the time spent in the chamber with a novel vs familiar mouse. Behavioral analyses were performed automatically by video tracking software (Ethovision Noldus) [39] All behavioral tests were performed in a specified behavioral suite under red light.

### Social dominance tube test

Animal social dominance was tested as previously described [41, 42] in a transparent Plexiglas tube measuring 30.5 cm in length and 3 cm diameter, a size just sufficient to permit one subject mouse to pass through without reversing direction. The tube was set on a plastic table in the designated behavioral suite and trials were manually recorded by a researcher blind to experimental groups. Animals were placed at opposite ends of the tube and released. A subject was declared the “winner” when its opponent backed out of the tube, with all 4 paws outside of the tube. The maximum test time allowed was 2 min. For 5 days, baseline social hierarchy was determined by once-daily tube tests for all animals within a five-mouse cage in a randomized order. In each cage, either the two most socially dominant or the two most subordinate mice were delivered HSV-ZFP189 TFs whereas the remaining cage-mates were delivered HSV-GFP. For the following 5 days the tube tests were repeated and wins for the HSV-ZFP189 TF treated mice versus all HSV-GFP treated mice was recorded to determine post-surgery social hierarchy. Social dominance was measured by calculating the percentage of wins in the tube test (number of wins/number of tests * 100).

### Five-trial social memory test

Five-trial social memory was tested as previously described [43] to determine ability to recognize novel versus familiar animals. Subjects were placed into the open arena (43 × 43 × 43 cm) with an empty wire cage (10 × 5 × 30 cm) at one side (interaction zone). Mice were given 2.5 min of habituation to explore the arena and then removed from the open arena. A novel C57BL/6 J male mouse was then placed within the wire cage (interaction zone) and the C57BL/6 J was placed back into the open arena and another 2.5 min were recorded (trial 1). The novel mouse was removed for 10 min. Subsequently, the same procedure was repeated three more times (i.e. subject mouse exposed to the familiar mouse, trials 2, 3 and 4). In trial 5, an unfamiliar male C57BL/6 J mouse was introduced to measure dishabituation. Time spent in the interaction zone for the first 30 s of each trial was measured. Behavioral analyses were performed automatically by video tracking software (Ethovision Noldus) [39]. All behavioral tests were performed in a specified behavioral suite under red light.

### Novel object recognition test

Novel object recognition was tested as previously described [44] In the first trial, the C57BL/6 J mouse was placed in the center of an open arena (43 × 43 × 43 cm) with two identical objects on opposite sides of the box, and was allowed to freely explore the objects for 5 min. In the second trial, (the object recognition memory testing phase) the mouse was allowed to explore objects for 5 min in the same open field box compromising one object used in the trial 2 (familiar object) and a new object replacing the second object used in trial 1 (the novel object.) A mouse is considered to be exploring an object when its nose is within 3 cm of the object. The arena was cleaned with 70% EtOH before a new C57BL/6 J mouse was placed in the arena and before the first trial. Times spent exploring the novel object, the familiar object, and both objects (total object exploration time) were collected. Furthermore, novel object discrimination index was calculated (time spent with novel object/total exploration time * 100.) Behavioral analyses were performed automatically by video tracking software (Ethovision Noldus) [39]. All behavioral tests were performed in a specified behavioral suite under red light.

### Microscopy

Three days following viral gene transfer, when HSV and GFP expression remained readily detectable, mice were transcardially perfused with 0.1 M sodium phosphate buffer, followed by 4% paraformaldehyde (PFA) in 0.1 M phosphate buffer. Brains were removed and postfixed in 4% PFA overnight at 4 °C. Following postfix, whole brains were stored in 15% sucrose in phosphate buffer with 0.05% sodium azide at 4 °C for 24 h, and then stored in 30% sucrose in phosphate buffer with 0.05% sodium azide at 4 °C until sectioning. Coronal sections at 40μm thick containing the PFC were then prepared on a Lecia VT1000S vibratome in 0.1 PBS. Sections were then mounted and coverslipped using ProLong Gold Antifade. Slides were kept at 4 °C until imaging in a light-blocking slide box until imaging. Sections were imaged and captured with Zeiss 880 Airyscan confocal microscope. Slices were imaged with a 63x oil-immersion magnification.

### Dendritic spine analysis

Images were captured on a Zeiss 880 Airyscan confocal microscope, and individual dendritic segments were focused on and scanned at 0.69-μm intervals along the z axis to obtain a z-stack. After capture, all images were deconvolved within the Zeiss Application Suite software. Analyses were performed on two-dimensional projection images using ImageJ (NIH). ~20 μm in length of dendrite were analyzed on captured neurons. For each group, 3-6 cells were analyzed in 3-5 different mice. We operationally divided spines into three categories; (1) mushroom-like spines were dendritic protrusions with a head diameter >0.5 μm or >2x the spine neck diameter; (2) stubby spines were dendritic protrusions with no discernable head and a length of ≤0.5 μm; and (3) thin/filopodia-like spines were dendritic protrusions with a length of >0.5 μm and head diameter <0.5 μm or no discernable head [45].

### Tissue preparation and RNA sequencing

Mice virally manipulated with HSV-ZFP189^NFD^, -ZFP189^WT^, or -ZFP189^VPR^ were used in RNAseq analysis. Mice were cervically dislocated and decapitated without anesthesia, and the brains were removed and sectioned into 1 mm coronal slices using brain matrices. Central tissue punch containing bilateral PFC (12 gauge; internal diameter, 2.16 mm) were snap frozen on dry ice and stored at -80 °C, as is routinely performed by our group [46, 47]. RNA was extracted and purified using RNeasy (Qiagen, Hilden, Germany), and total RNA was quantified with the Qubit RNA HS Assay Kit (Thermo Fisher Scientific, Waltham, MA). RNA quality control assays were performed on the TapeStation 4200 (Agilent, Santa Clara, CA), and the average RNA integrity number for all samples exceeded 8.6. Ribosomal RNA depletion and library preparation (Illumina Ribo-Zero) was performed, and RNA-seq was carried out at Genewiz with the following configuration: 2 × 150 paired-end reads on an Illumina (San Diego, CA) sequencing platform (HiSeq 2500) with a sequencing depth of ~101 million reads per sample (mean = 101 ± 23 million). Other overall sample sequencing statistics include the mean quality score (37.50 ± 0.01) and the percent of bases ≥30 (94.76 ± 0.02). Raw and processed RNAseq gene expression data are available via the Gene Expression Omnibus data (accession number GSE246064).

Sequence reads were trimmed to remove possible adapter sequences and nucleotides with poor quality using Trimmomatic v.0.36. The trimmed reads were mapped to the Mus musculus GRCm38 reference genome available on Ensembl using the STAR aligner v.2.5.2b. Unique gene hit counts were calculated by using featureCounts from the Subread package v.1.5.2. To estimate the expression levels of alternatively spliced transcripts (Supp. Fig. 6), the splice variant hit counts were extracted from the RNAseq reads mapped to the genome. Differentially spliced genes were identified for groups by testing for significant differences in read counts on exons (and junctions) of the genes using DEXSeq. Transposable element hit counts were calculated using featureCounts from the TEtranscripts package and were calculated separately [48]. After extraction of gene hit counts, the gene hit counts table was used for downstream differential expression analysis. Using DESeq2, a comparison of gene expression between groups of samples was performed. The Wald test was used to generate *p*-values and log_2_ fold changes. Genes with a Benjamini-Hochberg 5% false discovery rate (FDR) adjusted *p*-value < .05 were designated as DEGs for each comparison. Volcano plots with these DEGs were assembled using Graphpad Prism 10.

### DEG analysis approach

DEG tables from DESeq2 were imported into Rstudio for further analysis. For pattern identification in Venn diagrams and Gene Ontology (GO), cutoff criteria of nominal *p*-value < 0.05 and absolute log_2_ fold change >1.3 were applied for each comparison. For Venn diagrams, the significant DEGs were segregated by up- or downregulation by the sign of log_2_(FoldChange). WebGestalt [49] was used for GO over-representation analysis on gene lists (Benjamini-Hochberg adjusted false discovery rate <0.05). Outputs were graphically represented by fold enrichment and number of genes in each ontology using ggplot. Briefly, enriched ontology terms were ordered by FDR and plotted using ggplot in Rstudio, with each plot point colored by enrichment and sized by number of genes. Finally, entire DEG lists uploaded into IPA and weighted by fold change were analyzed against the Ingenuity Knowledge Base using both direct and indirect relationships [50]. Significantly activated and inhibited predicted upstream regulators were sorted by activation z-score and graphed into an activation heatmap using the ggplot package.

### Statistical analysis

All data was analyzed in GraphPad Prism 10. In all figures, results were expressed as mean ± standard error (S.E.M.) and were analyzed parametrically. Groups were analyzed with either a one-way ANOVA followed by a Bonferroni’s post-hoc test (Fig. 1C, D; Fig. 2D-G; Fig. 3B–D; Supp. Fig. 2B, C; Supp. Fig. 3A, B; Supp. Fig. 4B; Supp. Fig. 5B), a Student’s two-tailed t-test (Fig. 1E-G; Fig. 2F, G; Supp. Fig. 1; Supp. Fig. 2F, G), a two-way repeated measures ANOVA comparing main effect (Fig. 3I–L), or a two-way ANOVA followed by Bonferroni’s post-hoc test (Supp. Fig. 4B). *P*-value < 0.05 was considered statistically significant. Experimental sample sizes were guided by power analyses and previously published experiments from our group and others. Animals were randomly assigned to viral treatment conditions. Experimenters were blind to treatment and experimental analysis was performed by automated software (Ethovision, ImageJ).

### Supplementary information

Supplementary Figure 1

Supplementary Figure 2

Supplementary Figure 3

Supplementary Figure 4

Supplementary Figure 5

Supplementary Figure 6

Supplementary Figure 7

Supplementary Figure Legends

Supplementary Table 1 - 24h NFD vs VPR TEs and DEGs

Supplementary Table 2 - 24h NFD vs WT TEs and DEGs

Supplementary Table 3 - 4d NFD vs VPR DEGs

Supplementary Table 4 - 4d NFD vs WT DEGs

## Data Availability

Raw and processed RNAseq gene expression data are available via the Gene Expression Omnibus data (accession number GSE246064). Other data that support the findings from this study are available from the corresponding author upon request.

## References

[1] Liu H, Chang LH, Sun Y, Lu X, Stubbs L. Deep vertebrate roots for mammalian zinc finger transcription factor subfamilies. Genome Biol Evol. 2014. 10.1093/gbe/evu030 10.1093/gbe/evu030 PMC3971581 24534434

[2] Imbeault M, Helleboid PY, Trono D. KRAB zinc-finger proteins contribute to the evolution of gene regulatory networks. Nature. 2017. 10.1038/nature21683 10.1038/nature21683 28273063

[3] Playfoot CJ, Duc J, Sheppard S, Dind S, Coudray A, Planet E, et al. Transposable elements and their KZFP controllers are drivers of transcriptional innovation in the developing human brain. Genome Res. 2021. 10.1101/gr.275133.120 10.1101/gr.275133.120 PMC8415367 34400477

[4] Odeberg J, Røsok O, Gudmundsson GH, Ahmadian A, Roshani L, Williams C (1998). Cloning and characterization of ZNF189, a novel human Kruppel-like zinc finger gene localized to chromosome 9q22-q31. Genomics.

[5] Wolf G, de Iaco A, Sun MA, Bruno M, Tinkham M, Hoang D, et al. Krab-zinc finger protein gene expansion in response to active retrotransposons in the murine lineage. eLife. 2020. 10.7554/eLife.56337 10.7554/eLife.56337 PMC7289599 32479262

[6] Turelli P, Playfoot C, Grun D, Raclot C, Pontis J, Coudray A, et al. Primate-restricted KRAB zinc finger proteins and target retrotransposons control gene expression in human neurons. Sci. Adv. 2020. 10.1126/sciadv.aba3200 10.1126/sciadv.aba3200 PMC7455193 32923624

[7] Lorsch ZS, Hamilton PJ, Ramakrishnan A, Parise EM, Salery M, Wright WJ, et al. Stress resilience is promoted by a Zfp189-driven transcriptional network in prefrontal cortex. Nat Neurosci. 2019. 10.1038/s41593-019-0462-8 10.1038/s41593-019-0462-8 PMC6713580 31427770

[8] Kennedy DP, Adolphs R. The social brain in psychiatric and neurological disorders. Trends Cogn Sci. 2012. 10.1016/j.tics.2012.09.006 10.1016/j.tics.2012.09.006 PMC3606817 23047070

[9] Afifi TO, Enns MW, Cox BJ, Asmundson GJG, Stein MB, Sareen J, et al. Population attributable fractions of psychiatric disorders and suicide ideation and attempts associated with adverse childhood experiences. Am J Public Health. 2008. 10.2105/AJPH.2007.120253 10.2105/AJPH.2007.120253 PMC2374808 18381992

[10] Najafabadi HS, Mnaimneh S, Schmitges FW, Garton M, Lam KN, Yang A (2015). C2H2 zinc finger proteins greatly expand the human regulatory lexicon. Nat Biotechnol.

[11] Hamilton PJ, Burek DJ, Lombroso SI, Neve RL, Robison AJ, Nestler EJ (2018). Cell-type-specific epigenetic editing at the fosb gene controls susceptibility to social defeat stress. Neuropsycho Pharmacol.

[12] Heller EA, Hamilton PJ, Burek DD, Lombroso SI, Peña CJ, Neve RL (2016). Targeted epigenetic remodeling of the Cdk5 gene in nucleus accumbens regulates cocaine- and stress-evoked behavior. J Neurosci.

[13] Neve RL, Neve KA, Nestler EJ, Carlezon WA (2005). Use of herpes virus amplicon vectors to study brain disorders. Biotechniques.

[14] Filiano AJ, Xu Y, Tustison NJ, Marsh RL, Baker W, Smirnov I, et al. Unexpected role of interferon-γ 3 in regulating neuronal connectivity and social behaviour. Nature. 2016. 10.1038/nature18626 10.1038/nature18626 PMC4961620 27409813

[15] Leonardi I, Gao IH, Lin WY, Allen M, Li XV, Fiers WD (2022). Mucosal fungi promote gut barrier function and social behavior via Type 17 immunity. Cell.

[16] Reed MD, Yim YS, Wimmer RD, Kim H, Ryu C, Welch GM (2020). IL-17a promotes sociability in mouse models of neurodevelopmental disorders. Nature.

[17] De Hardy TJ, Thorball CW, Forey R, Planet E, Duc J, Khubieh B, et al. Genetic features and genomic targets of human KRAB-zinc finger proteins. Genomics. 2023. 10.1101/2023.02.27.530095 10.1101/gr.277722.123 PMC10547255 37730438

[18] Sundaram V, Wysocka J (2020). Transposable elements as a potent source of diverse cis-regulatory sequences in mammalian genomes. Philos Trans R Soc Lond B Biol Sci.

[19] Chuong EB, Elde NC, Feschotte C (2016). Regulatory evolution of innate immunity through co-option of endogenous retroviruses. Science.

[20] Ye M, Goudot C, Hoyler T, Lemoine B, Amigorena S, Zueva E (2020). Specific subfamilies of transposable elements contribute to different domains of T lymphocyte enhancers. Proc Natl Acad Sci USA.

[21] Teague CD, Picone JA, Wright WJ, Browne CJ, Silva GM, Futamura R (2023). CREB binding at the Zfp189 promoter within medium spiny neuron subtypes differentially regulates behavioral and physiological adaptations over the course of cocaine use. Biol Psychiatry.

[22] Lardner CK, van der Zee Y, Estill MS, Kronman HG, Salery M, Cunningham AM (2021). Gene-targeted, CREB-mediated induction of ΔFosB controls distinct downstream transcriptional patterns within D1 and D2 medium spiny neurons. Biol Psychiatry.

[23] van der Zee YY, Lardner CK, Parise EM, Mews P, Ramakrishnan A, Patel V (2022). Sex-specific role for SLIT1 in regulating stress susceptibility. Biol Psychiatry.

[24] Issler O, van der Zee YY, Ramakrishnan A, Wang J, Tan C, Loh Y-HE (2020). Sex-specific role for the long non-coding RNA LINC00473 in depression. Neuron.

[25] Mello CV, Vicario DS, Clayton DF (1992). Song presentation induces gene expression in the songbird forebrain. Proc Natl Acad Sci USA.

[26] Dong S, Clayton DF (2008). Partial dissociation of molecular and behavioral measures of song habituation in adult zebra finches. Genes Brain Behav.

[27] Burmeister SS, Jarvis ED, Fernald RD (2005). Rapid behavioral and genomic responses to social opportunity. PLoS Biol.

[28] Weaver ICG, Cervoni N, Champagne FA, D’Alessio AC, Sharma S, Seckl JR (2004). Epigenetic programming by maternal behavior. Nat Neurosci.

[29] Zhao Y, Oreskovic E, Zhang Q, Lu Q, Gilman A, Lin YS, et al. Transposon-triggered innate immune response confers cancer resistance to the blind mole rat. Nat. Immunol. 2021. 10.1038/s41590-021-01027-8 10.1038/s41590-021-01027-8 PMC8488014 34556881

[30] Oka K, Fujioka S, Kawamura Y, Komohara Y, Chujo T, Sekiguchi K (2022). Resistance to chemical carcinogenesis induction via a dampened inflammatory response in naked mole-rats. Commun Biol.

[31] Lin T, Buffenstein R (2021). The unusual immune system of the naked mole-rat. Adv Exp Med Biol.

[32] Kashash Y, Smarsh G, Zilkha N, Yovel Y, Kimchi T (2022). Alone, in the dark: the extraordinary neuroethology of the solitary blind mole rat. Elife.

[33] Schulze-Makuch D (2019). The naked mole-rat: an unusual organism with an unexpected latent potential for increased intelligence?. Life Basel.

[34] Snyder-Mackler N, Sanz J, Kohn JN, Brinkworth JF, Morrow S, Shaver AO (2016). Social status alters immune regulation and response to infection in macaques. Science.

[35] Snyder-Mackler N, Sanz J, Kohn JN, Voyles T, Pique-Regi R, Wilson ME (2019). Social status alters chromatin accessibility and the gene regulatory response to glucocorticoid stimulation in rhesus macaques. Proc Natl Acad Sci USA.

[36] Skalka AM (2014). Retroviral DNA transposition: themes and variations. Microbiol Spectr.

[37] Hamilton PJ, Lim CJ, Nestler EJ, Heller EA (2018). Viral expression of epigenome editing tools in rodent brain using stereotaxic surgery techniques. Methods Mol Biol.

[38] Golden SA, Covington HE, Berton O, Russo SJ (2011). A standardized protocol for repeated social defeat stress in mice. Nat Protoc.

[39] Noldus LP, Spink AJ, Tegelenbosch RA (2001). EthoVision: a versatile video tracking system for automation of behavioral experiments. Behav Res Methods Instrum Comput.

[40] Pellow S, File SE (1986). Anxiolytic and anxiogenic drug effects on exploratory activity in an elevated plus-maze: a novel test of anxiety in the rat. Pharm Biochem Behav.

[41] Lindzey G, Winston H, Manosevitz M (1961). Social dominance in inbred mouse strains. Nature.

[42] Han KA, Yoon TH, Shin J, Um JW, Ko J. Differentially altered social dominance- and cooperative-like behaviors in Shank2- and Shank3-mutant mice. Mol Autism. 2020. 10.1186/s13229-020-00392-9 10.1186/s13229-020-00392-9 PMC7602353 33126897

[43] Hitti FL, Siegelbaum SA (2014). The hippocampal CA2 region is essential for social memory. Nature.

[44] Antunes M, Biala G (2012). The novel object recognition memory: neurobiology, test procedure, and its modifications. Cogn Process.

[45] Wright WJ, Graziane NM, Neumann PA, Hamilton PJ, Cates HM, Fuerst L (2020). Silent synapses dictate cocaine memory destabilization and reconsolidation. Nat Neurosci.

[46] Hamilton PJ, Chen EY, Tolstikov V, Peña CJ, Picone JA, Shah P (2020). Chronic stress and antidepressant treatment alter purine metabolism and beta oxidation within mouse brain and serum. Sci Rep..

[47] Townsend EA, Kim RK, Robinson HL, Marsh SA, Banks ML, Hamilton PJ (2021). Opioid withdrawal produces sex-specific effects on fentanyl-vs.-food choice and mesolimbic transcription. Biol Psychiatry Glob Open Sci.

[48] Jin Y, Tam OH, Paniagua E, Hammell M (2015). TEtranscripts: a package for including transposable elements in differential expression analysis of RNA-seq datasets. Bioinformatics.

[49] Liao Y, Wang J, Jaehnig EJ, Shi Z, Zhang B (2019). WebGestalt 2019: gene set analysis toolkit with revamped UIs and APIs. Nucleic Acids Res.

[50] Krämer A, Green J, Pollard J, Tugendreich S (2014). Causal analysis approaches in ingenuity pathway analysis. Bioinformatics.

