# Supplementary figures and images for: A zinc finger transcription factor enables social behaviors while controlling transposable elements and immune response in prefrontal cortex
Source: Transl Psychiatry. 2024 Jan 25;14:59. doi: 10.1038/s41398-024-02775-5 (PMC10810849; doi:10.1038/s41398-024-02775-5)

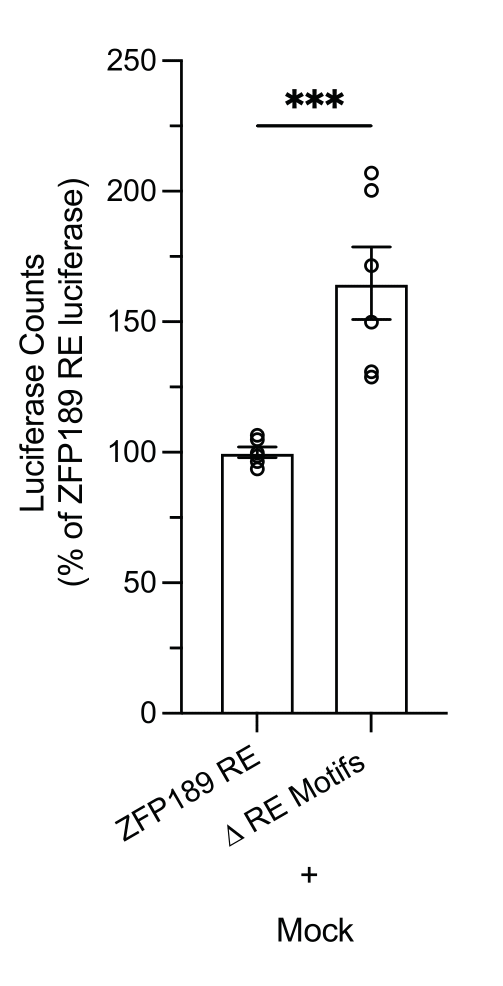

Supplement: Supplementary file 1 — Supplementary Figure 1 [file 41398_2024_2775_MOESM1_ESM.png]

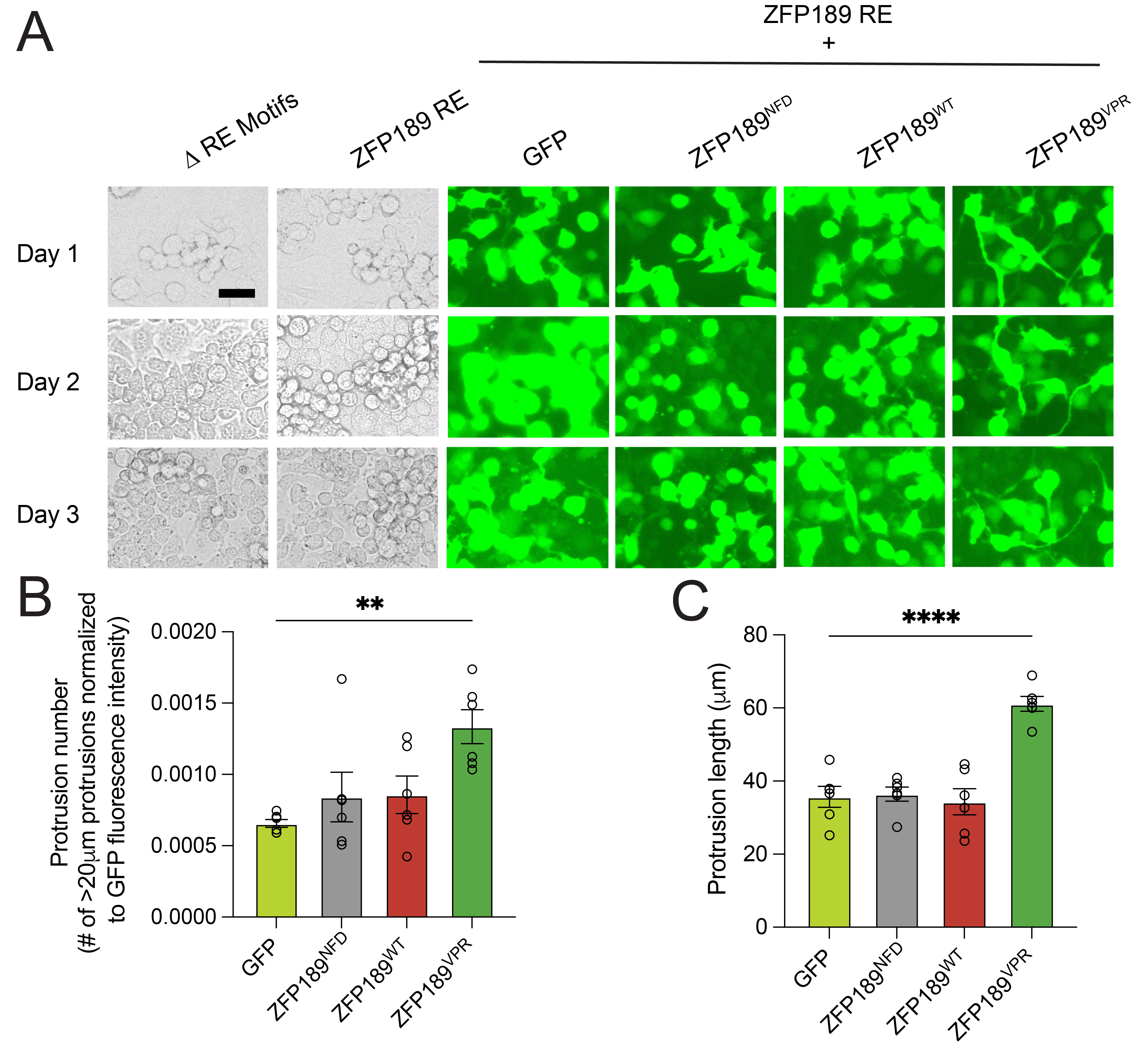

Supplement: Supplementary file 2 — Supplementary Figure 2 [file 41398_2024_2775_MOESM2_ESM.png]

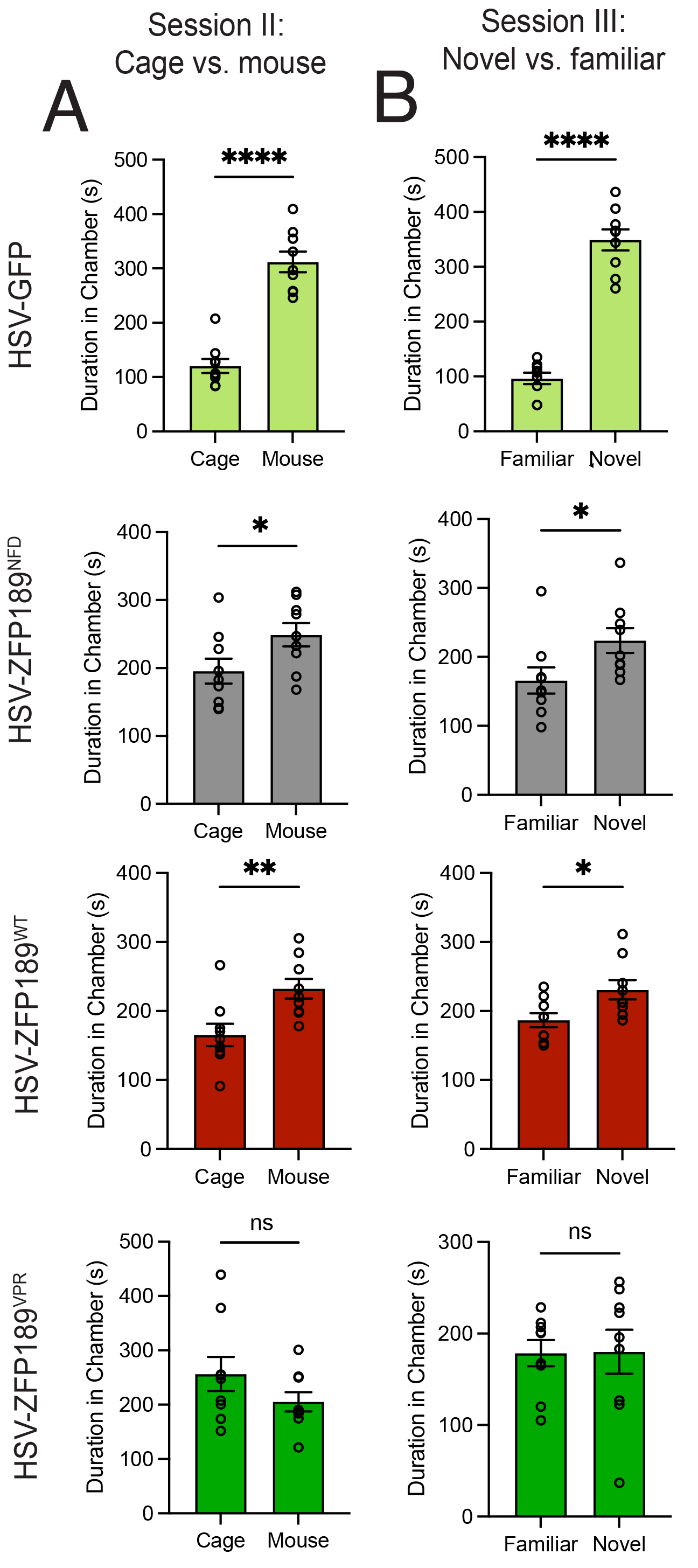

Supplement: Supplementary file 3 — Supplementary Figure 3 [file 41398_2024_2775_MOESM3_ESM.png]

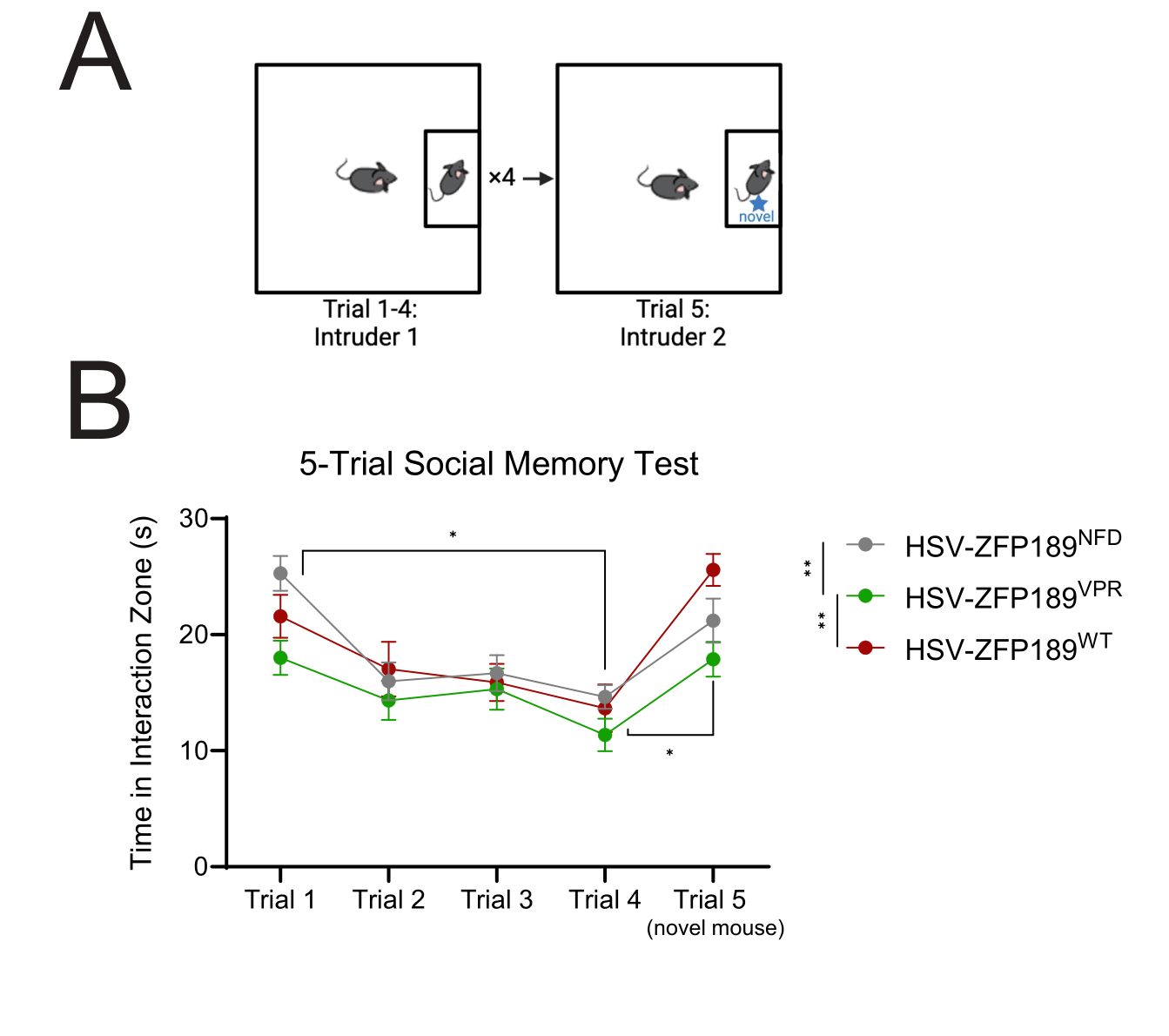

Supplement: Supplementary file 4 — Supplementary Figure 4 [file 41398_2024_2775_MOESM4_ESM.png]

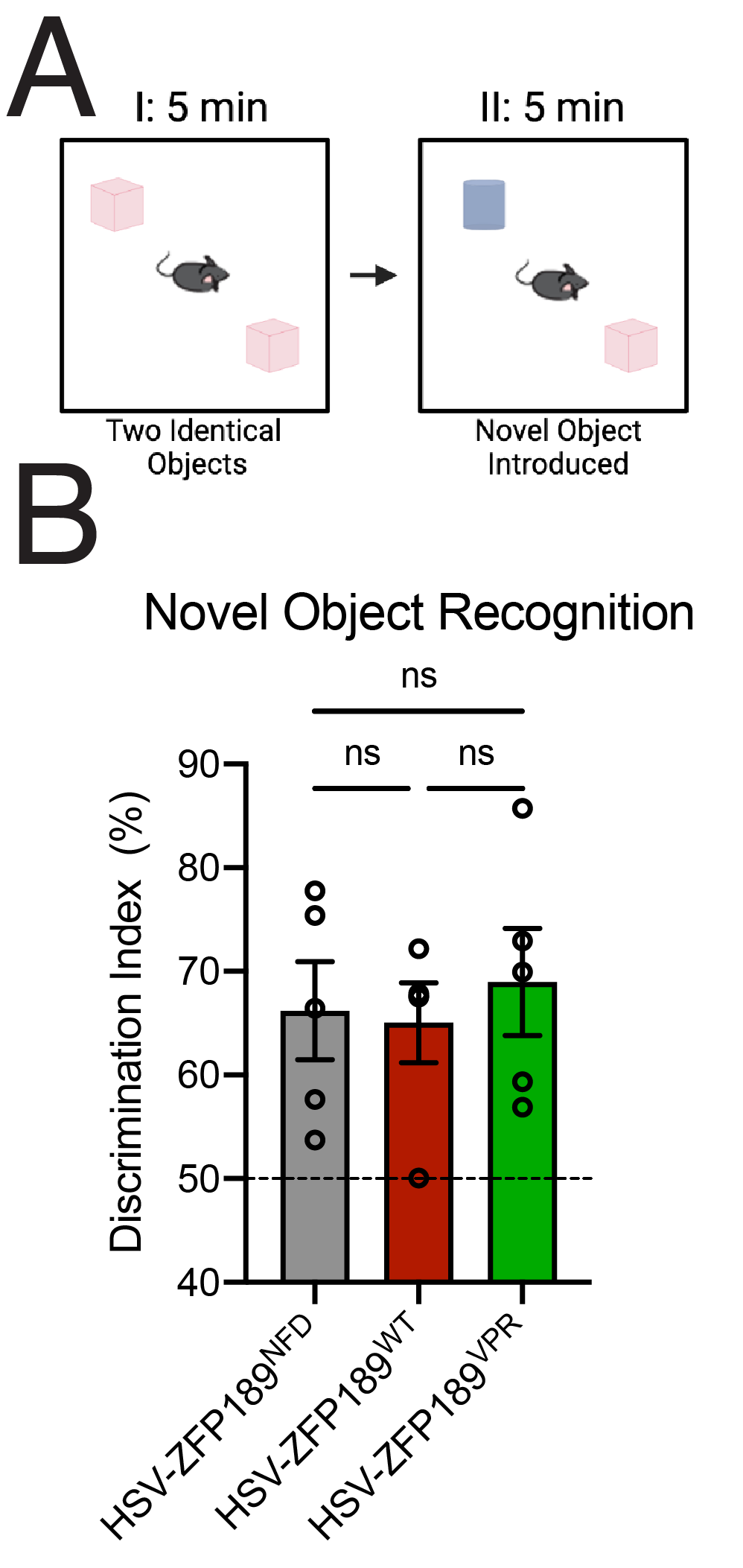

Supplement: Supplementary file 5 — Supplementary Figure 5 [file 41398_2024_2775_MOESM5_ESM.png]

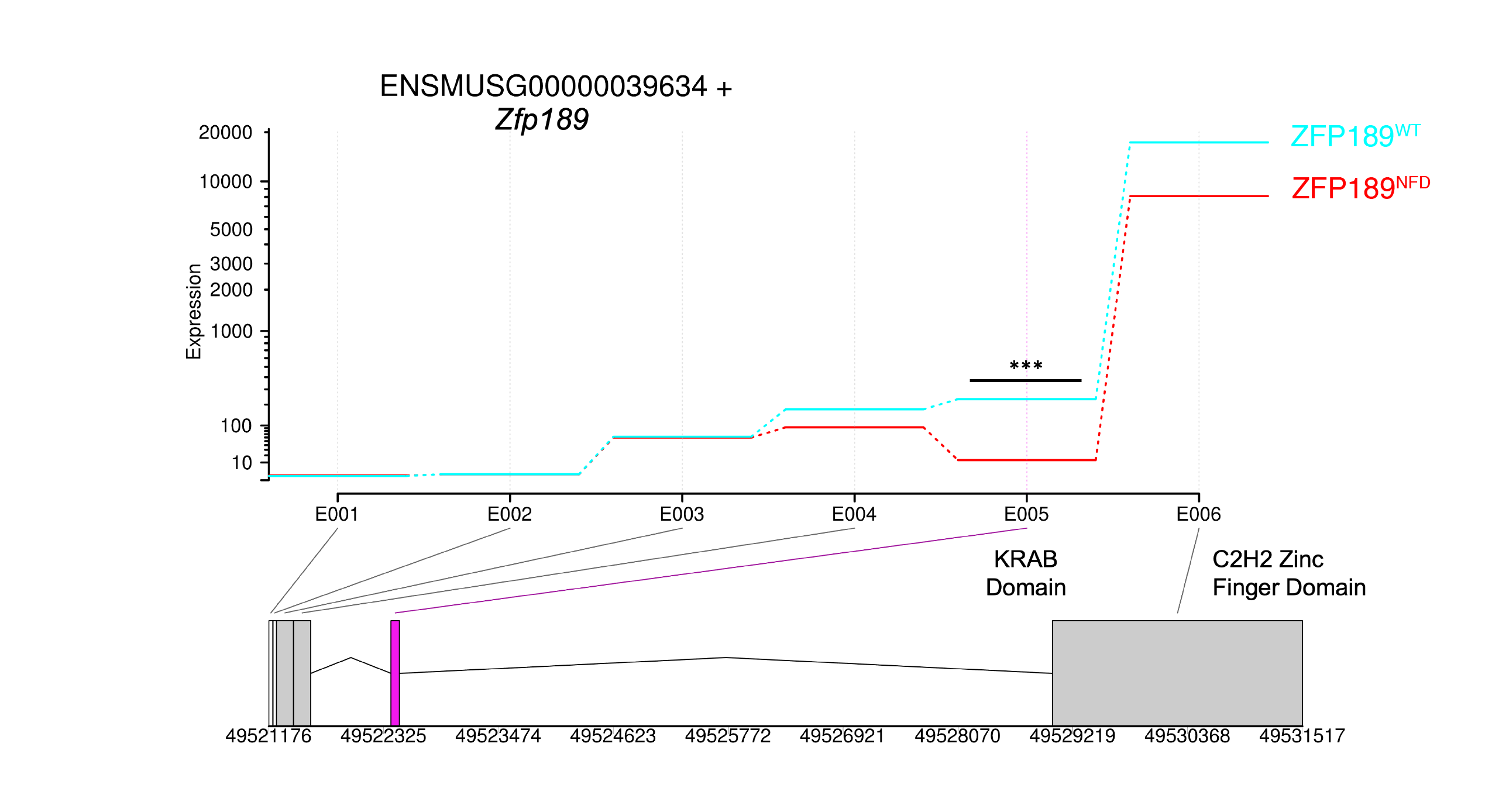

Supplement: Supplementary file 6 — Supplementary Figure 6 [file 41398_2024_2775_MOESM6_ESM.png]

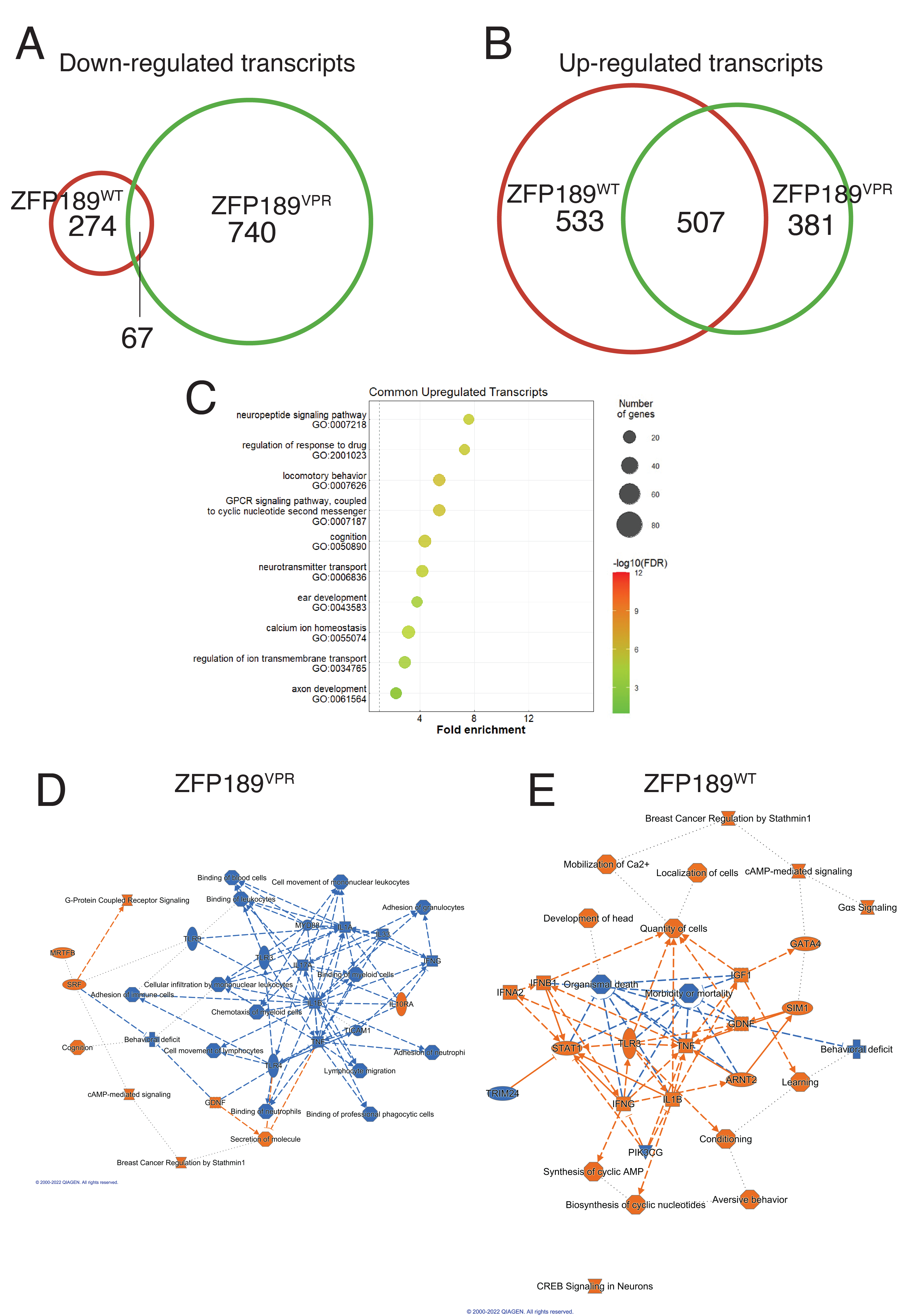

Supplement: Supplementary file 7 — Supplementary Figure 7 [file 41398_2024_2775_MOESM7_ESM.png]
