# Supplementary material for: A zinc finger transcription factor enables social behaviors while controlling transposable elements and immune response in prefrontal cortex
Source: Transl Psychiatry. 2024 Jan 25;14:59. doi: 10.1038/s41398-024-02775-5 (PMC10810849; doi:10.1038/s41398-024-02775-5)
Supplement: Supplementary file 8 — Supplementary Figure Legends [file 41398_2024_2775_MOESM8_ESM.docx]

Supplementary Figure Legends

**Supplemental Figure 1: Transfecting ZFP189 luciferase reporters either possessing or lacking the ZFP189 DNA Response Element (RE) reveal the natural gene-repressive functions of ZFP189 endogenously expressed in N2a cells.** N2a cells transfected with luciferase reporter target plasmids possessing (ZFP189 RE) or lacking (∆ RE motifs) the ZFP189 RE promoter sequence uncovers the endogenous repressive action of ZFP189 in the mouse neuronal N2a cells. Removing ZFP189 DNA binding domains in the ZFP189 null condition increases luciferase expression, revealing a release of a repressive effect mediated by an N2a endogenously expressed protein that binds ZFP189 REs. Data normalized to ZFP189 RE condition, by experiment. Two-tailed, unpaired Student’s *t*-test; **** p-value < 0.001. n = 6 per condition.

**Supplemental Figure 2: Transfecting N2a cells with ZFP189^VPR^ increases morphological complexity by increasing the number and length of cellular protrusions. (A)** Microscopy on N2a cells transfected with only luciferase reporter plasmids (phase contrast; no plasmid GFP cassette in luciferase plasmids) or ZFP189 RE luciferase plasmid alongside synthetic ZFP189 TFs (fluorescent microscopy; GFP driven by separate CMV promoter in TF plasmids) for three days following the transfection reaction. Scale bar is 20 μm. **(B)** N2a cells expressing ZFP189^VPR^ have more cellular protrusions. On day 2, in each GFP+ condition, the total number of cellular protrusions in GFP+ cells measuring greater than 20 μm was quantified in each well and normalized to GFP fluorescence intensity for that well, to account for transfection efficiency. One-way ANOVA followed by Bonferroni’s multiple comparisons test relative to GFP condition. ** p-value < 0.01; n = 6 per condition. **(C)** N2a cells expressing ZFP189^VPR^ have longer cellular protrusions. On day 2, the length of each cellular outgrowth was measured and averaged per well. One-way ANOVA followed by Bonferroni’s multiple comparisons test relative to GFP condition. **** p-value < 0.0001; n = 6 per condition.

**Supplemental Figure 3: Inverting the natural transcriptional control of ZFP189 in the PFC with HSV-ZFP189^VPR^ impairs social function in female mice (A)** In session II, when given the choice between interacting with an empty cage or a novel mouse, HSV-GFP, HSV-ZFP189^NFD^, and HSV-ZFP189^WT^ treated female mice spend significantly more time interacting with the novel mouse, whereas HSV-ZFP189^VPR^ treated mice do not. Unpaired two-tailed Student’s *t*-test. **** p-value < 0.0001, ** p-value < 0.005, * p-value < 0.05, ns p-value > 0.05. **(B)** Subjecting these same animals to session III wherein female test mice are given a choice between interacting with the caged mouse from the previous round (Familiar) or a novel mouse, HSV-GFP, HSV-ZFP189^NFD^, and HSV-ZFP189^WT^ treated mice again spend more time interacting with the novel mouse, whereas HSV-ZFP189^VPR^ treated mice do not. * p-value < 0.05, ns p-value > 0.05. n = 9 mice (HSV-GFP), n = 9 mice (HSV-ZFP189^NFD^), n = 9 mice (HSV-ZFP189^WT^), n = 9 mice (HSV-ZFP189^VPR^).

**Supplemental Figure 4: No viral treatment condition impacts social recognition or social memory. (A)** Schematic of five-trial social memory paradigm. Mice manipulated with our synthetic ZFP189 TFs were placed in an arena with an unfamiliar target mouse for a 2.5min trial duration. This is repeated with the same test mouse and target mouse for trials 1 through 4. In the 5^th^ trial, a novel mouse was introduced as the target mouse. Total time interacting with target mouse during the first 30 seconds of the trial is shown for each trial. **(B)** Mice in all groups show habituation to the familiarity of the test mouse in trials 1-4. All mice increase time spent socializing with the novel mouse in trial 5, indicating typical social memory in all groups. Two-way ANOVA followed by Bonferroni’s multiple comparisons test. 1 vs. 4, **** p-value < 0.0001; 4 vs. 5, * p-value < 0.05 (HSV-ZFP189^NFD^), 1 vs. 4, * p-value < 0.05; 4 vs. 5, **** p-value < 0.0001 (HSV-ZFP189^WT^), 1 vs. 4, * p-value < 0.05; 4 vs. 5, * p-value < 0.05 (HSV-ZFP189^VPR^). Additionally, over all tests, HSV-ZFP189^VPR^ treated mice interacted with social target mice less than HSV-ZFP189^NFD^ and HSV-ZFP189^WT^ treated mice. Two-way ANOVA followed by Bonferroni’s multiple comparisons test for viral main effect over all trials. ** p-value < 0.005. n = 14 mice (HSV-ZFP189^NFD^), n = 12 mice (HSV-ZFP189^WT^), n = 15 mice (HSV-ZFP189^VPR^).

**Supplemental Figure 5: No viral treatment condition impairs novel object recognition. (A)** Schematic showing novel object recognition paradigm. Mice manipulated with our synthetic ZFP189 TFs were placed in arena with two identical objects on opposite sides of the box, and were allowed to freely explore the objects for five minutes. In the second trial, the mouse was allowed to explore objects for 5 minutes in the same arena compromising one object used in the trial 2 (familiar object) and a new object replacing the second object used in trial 1 (the novel object.)  **(B)** Represents the discrimination index percentage (DI) for each experimental group. DI = time spend with novel object/total exploration time * 100. All experimental groups were able to recognize novel object, indicated by DI > 50, and no viral treatment group performed significantly differently. One-way ANOVA followed by Bonferroni’s multiple comparisons test. ns p-value > 0.05.

**Supplemental Figure 6: RNAseq validates differential PFC expression of the *Zfp189* exon encoding the KRAB domain.** In the RNAseq dataset associated with Figure 4D (i.e. the ZFP189^NFD^ vs. ZFP189^WT^ comparison four days post viral delivery), reads aligning specifically to the *Zfp189* exon encoding the KRAB domain is significantly diminished in the ZFP189^NFD^-treated mice. This is a useful validation that we can detect the intended absence of the functional KRAB domain in ZFP189^NFD^-treated mice. With this same analysis, no significant difference was detected in the dataset associated with Figure 4C (i.e. the ZFP189^NFD^ vs. ZFP189^VPR^ comparison four days post viral delivery), since both conditions lacked the endogenous KRAB domain (not shown).

**Supplemental Figure 7: Synthetic ZFP189 TFs have converging up-regulation of PFC transcripts involved in synaptic neurotransmission.** Venn diagrams comparing down- (**A**) and up-regulated (**B**) transcripts regulated by each ZFP189 TF. Significance cutoff set to nominal *p* value < .05 and absolute log_2_ fold change > 1.3 to enable broad pattern recognition. **C**) Gene-ontology analysis of the 507 commonly up-regulated transcripts from panel B. Ingenuity pathway analysis of ZFP189^VPR^-regulated (**D**) and ZFP189^WT^-regulated (**E**) transcripts.
